# Integrated transcriptomics and metabolomics to explore the mechanisms of *Elaeagnus mollis* diels seed viability decline

**DOI:** 10.1186/s12864-025-11483-3

**Published:** 2025-04-02

**Authors:** Ren Ruifen, Guo Jiayi, Ji Zhe, Du Shuhui, Yang Xiuyun

**Affiliations:** College of Forestry, Shanxi Agricultural University, Taigu, 030801 China

**Keywords:** *Elaeagnus mollis*, Seed, Viability decline, Transcriptomics, Metabolomics, Antioxidant systems

## Abstract

*Elaeagnus mollis* Diels, is a rare and endangered woody plant endemic to China, which is listed on the IUCN Red List. In the natural state, the viability of its seeds declines very rapidly, which is the key to its endangered status, but the mechanism of *E. mollis* seed viability decline is still unclear. In order to explore the physiological and molecular mechanism of viability decline of *E. mollis* seeds, this study used fresh seeds as a control to compare and analyze the changes of seed vitality, antioxidant system, transcription and metabolomics, when seeds were stored for 1 and 3 months at room temperature. The viability of *E. mollis* seed decreased continuously after 1 month and 3 months of storage. The activities of superoxide dismutase (SOD), monodehydroascorbate reductase (MDHAR), ascorbate (AsA), and glutathione (GSH) decreased significantly, while catalase (CAT) activity increased gradually during the decline of seed viability. Transcriptomic results showed that a total of 801 differentially expressed genes (DEGs) were identified between fresh and 1-month-stored seeds, while 1,524 were identified between fresh and 3-month-stored seeds. Among them, the expression of *CAT*, *MDHAR*, *GSH* and *GR* were consistent with the results of physiological indicators. Moreover, *WRKY*, *C3H*, *bZIP*, *B3*, *bHLH*, *NAC* and *AP2 / ERF-ERF* transcription factors are important in regulating seed viability. Metabolomics results showed that the types of differential accumulated metabolites (DAMs) during viability decline were mainly flavonoids, amino acids and derivatives, and phenolic acids. The combined analysis results of transcriptomics and metabolomics further showed that DEGs and DAMs associated with viability were co-enriched in flavonoid biosynthesis and tryptophan metabolism pathways. Also identified were 22 key antioxidant genes, including *CAT*, *ALDH*, *CHS* and *C4H*, which were identified as participating in the changes of seed viability. This also illustrated that the metabolic pathways of flavonoid biosynthesis and tryptophan metabolism were involved in regulating the decline of seed viability by acting on the antioxidant system. These findings provide new insights into the mechanism of seed viability decline of *E. mollis*.

**Supplementary Information:**

The online version contains supplementary material available at 10.1186/s12864-025-11483-3.

## Introduction

Seeds, important carriers of genetic material, are the material basis for agricultural production and the development of plant species [1]. Seed quality determines to a large extent the growth and development of the plant and its yield. Seed viability is one of the most important indicators of quality, so maintaining and improving seed viability is important for seedling propagation and conservation of genetic diversity [2]. Effective seed conservation is the key to reducing the degradation of seed varieties, especially for some rare and endangered species, where effective conservation is particularly important [3–4]. However, in practical application, the majority of seeds are unable to be used immediately after collection. With the prolongation of the storage time, the seed are subjected to a combined effect of internal and external factors, which ultimately lead to an irreversible and unavoidable decline in viability [5]. Concurrently, in the natural state, the decline of seed viability represents a significant limiting factor for the regeneration and reproduction of plant populations.

The decline in seed viability is accompanied by a series of morphological, physiological, biochemical, and genetic changes, such as membrane lipid peroxidation [6–7], protein denaturation [8–9], gene expression disorder and nucleic acid degradation [10–11]. Current national and international studies on the decline in seed viability, have concentrated on investigating seed preservation from physiological and biochemical perspectives. Reactive oxygen species (ROS) are oxygen-containing atoms or groups of atoms with active chemical properties, including substances such as superoxide anion radicals (O_2_^−^), hydroxyl radicals (·OH), and hydrogen peroxide (H_2_O_2_) [12]. Antioxidant enzymes play a pivotal role in the ROS scavenging system. Superoxide dismutase (SOD) is an enzyme that catalyzes the disproportionation of superoxide anion radicals to oxygen (O_2_) and H_2_O_2_. Catalase (CAT) and ascorbate peroxidase (APX) are the most crucial H_2_O_2_ scavenging enzymes, catalyzing the conversion of H_2_O_2_ to water (H_2_O) and O_2_ [13]. Scholars have found that within the seed, ROS at low levels will not cause damage to cells [14]. However, with the decline of antioxidant enzyme activity and non-enzymatic substances, ROS continue to accumulate, which can damage the cell lipids, proteins and DNA and other biological macromolecules, causing oxidative stress, and oxidative damage to the cell, resulting in cell death, and ultimately leading to a decline in the viability of the seeds [15–16]. It is undeniable that the viability of all seeds will eventually be lost, even when they are preserved under optimal conditions [17]. Although much progress has been made in this area of research, effective preservation can only mitigate this decline in seed viability to a certain extent, thereby extending seed longevity, and cannot completely inhibit the decline in viability [18].

The field of transcriptomics is dedicated to the study of gene function and structure, which plays a key role in the development of living organisms [19]. In *Arachis hypogaea* seeds, 583 differentially expressed genes were identified between the treated (high viability and low viability) and 860 in the untreated seeds, which mainly regulated seed viability through cell wall remodeling, transcriptional regulation, and oxidative stress [20]. In *Chenopodium quinoa* seeds, key candidate genes, such as *CHS*, *CHI*, *AACT*, *ENO1*, *IDH*, *NADP-ME* and *HAO2L*, play a crucial role in the viability changes during seed storage [21]. Metabolites are the basis of organism phenotypes, and metabolomics reflects changes closer to the seed phenotype than other omics techniques [22]. In *Arabidopsis thaliana* and *Triticum aestivum* metabolomics studies, primary and secondary metabolites changes were found to significantly affect seed viability, and these metabolites mainly included quercetin, kaempferol, DL methionine and other substances [23–24]. However, the gene level and metabolite differences affecting *E. mollis* seed viability are still unknown.

*E. mollis* is an endemic Chinese woody plant with a variety of uses, including medicinal, edible, and ecological applications. It is one of the plant species that survived the glaciation in the fourth century, and is only found in localized areas of Shanxi and Shaanxi, China, with a very small distribution range [25–26]. As early as the 1980s, it was included in the national list of second-class rare and endangered protected plants and subsequently included in the International Union for Conservation of Nature Red List (Version 2018-2, https://www.iucnredlist.org/en) [27]. Previous studies demonstrated that in the natural state, the lifespan of *E. mollis* seeds are generally short and they are prone to inactivation when stored in vitro, rendering it unable to form a permanent seed bank. This constrains population renewal, thereby placing the species in a state of endangerment [28–29]. The majority of existing research on *E. mollis* concentrated on the investigation of community characteristics and genetic diversity, the development of rapid propagation systems, the examination of seed germination characteristics, and the purification and analysis of tissue inclusions [30–32]. While it is evident that low seed viability is one of the main reasons for the endangerment of the germplasm resources of *E. mollis*, the existing studies have not addressed the mechanism of seed viability decline.

In this study, fresh *E. mollis* seeds were used as a control to investigate the critical phases (1 month and 3 months of storage) of viability decline during the room temperature storage. We systematically analyzed the changes of seed viability, physiological levels, transcriptome, and metabolome. By integrating the data on antioxidant system, DEGs, DAMs, and key metabolic pathways in *E. mollis* seeds at different storage periods, to identify the key regulatory genes and core metabolic networks associated with viability decline, which will reveal elucidate the physiological and molecular mechanisms underlying of *E. mollis* seed viability decline, then providing a foundation for exploration of seed aging processes.

## Materials and methods

### Plant materials

The seeds of *E. mollis* used in this study, which were collected from 6-year-old trees at seed maturity in August 2023 at No. 1 Base of the National Innovation Alliance of *E. mollis* Industry of Yicheng, Shanxi, China. The individual plants were identified by plant taxonomists (Du Shuhui) of Shanxi Agricultural University, and the accuracy was confirmed. The related specimens are stored in the Herbarium, Institute of Botany, CAS (https://www.cvh.ac.cn/spms/detail.php?id=ef64ee21), and the deposition number was PE 01015651 (cvh.ac.cn), and it was collected in the Specimen Museum of Shanxi Agricultural University. The collected mature seeds were naturally air-dried and stored in the laboratory in the natural state. Storage temperature 25 ± 5℃, Dry humidity 45% ± 2%. The tissue sampled was the seed kernel, The samples were fresh seeds (CK), seeds stored for 1 month and 3 months at room temperature. After storage, the samples were immediately placed in liquid nitrogen (LN) and stored it in a -80 ℃ refrigerator before analysis. Each sample had three biological replicates, with ten seeds per replicate.

### Determination of seed viability

We selected 30 seeds each of control, 1 m stored, 3 m stored, 5 m stored and 7 m stored to determine their viability. The seeds were soaked in distilled water for 12 h before being peeled and divided into two halves along the longitudinal section of the embryo. The divided seeds were placed in 0.3% (*w*/*v*), 25 ml TTC staining solution, and stained at 25℃ in the dark for 12 h [33]. The stained seeds were taken out and excess water was pipetted off, then 5 stained seeds were randomly selected for quantitative TTCH analysis. The seeds were placed in a centrifuge tube with 2 mL of anhydrous ethanol and heated in a water bath at 80 °C for 1 h. After the samples from which the red material was extracted were cooled, the absorbance values of the extracts were determined using a spectrophotometer at 490 nm. Each sample had three biological replicates and the results were averaged.

### Determination of seed physiological indicators

O_2_^−^ content was determined by the hydroxylamine oxidation method [34]. The seeds (0.05 g) were homogenized with 1 ml extraction solution on ice and centrifuged at 10,000 rpm/min for 20 min at 4℃. Then, the supernatant was kept and phosphate buffer solution (PBS, included 1% polyvinylpyrrolidone, 20 mmol/L ethylenediaminete traacetic acid disodium salt and 2 mmol/L hydroxylamine hydrochloride, PH = 7.8), hydroxylamine hydrochloride, sulfanilic acid and α-naphthylamine were added sequentially to determine the results. Each sample had three biological replicates and the results were averaged.

H_2_O_2_ and ·OH contents were determined by hydrogen peroxide assay kit (Cat no., A064-1-1) and hydroxyl radical assay kit (Cat no., A018-1-1) from Nanjing Jiancheng Biotechnology Institute, China. The specific steps were followed by the instructions. Each sample had three biological replicates and the results were averaged.

SOD and CAT activity were determined by the method of Mathew with slight modifications [35]. The seeds (0.05 g) were homogenized with 1.2 ml PBS (included 5 mmol/L sodium dihydrogen phosphate and 115 mmol/L disodium hydrogen phosphate, PH = 7.8) on ice and centrifuged at 10,000 rpm/min for 15 min at 4℃. Then, the supernatant was taken and SOD and CAT reaction solution added to determine the results. Each sample had three biological replicates and the results were averaged.

The ascorbic acid (AsA) content was determined by the method of Kampfenkel with slight modifications [36]. The seeds (0.1 g) were homogenized with 2 ml 10% TCA on ice and centrifuged at 10,000 rpm/min for 15 min at 4℃. Then, the supernatant was taken, and sodium dihydrogen phosphate solution, distilled water, trichloroacetic acid, phosphoric acid solution, 2.2-dipyridyl solution and ferric chloride solution were added sequentially to determine the results. Each sample had three biological replicates and the results were averaged.

The glutathione (GSH) content was determined by Griffith’s method [37]. The seeds (0.1 g) were homogenized with 1.5 ml 10% TCA on ice and centrifuged at 10,000 rpm/min for 15 min at 4℃. Then, the supernatant was taken, and distilled water, PBS (included 57 mmol/L disodium hydrogen phosphate and 7 mmol/L potassium dihydrogen phosphate, PH = 7.7) and 2-nitrobenzene sulfonic acid solution were added sequentially to determine the results. Each sample had three biological replicates and the results were averaged.

APX activity was determined by the method of Ren [34]. The seeds (0.1 g) were homogenized with 1 ml extraction solution on ice and centrifuged at 10,000 rpm/min for 15 min at 4℃. Then, the supernatant was taken, and PBS (included 2 mmol/L ascorbic acid and 5 mmol/L ethylenediamine tetraacetic acid, PH = 7.8), AsA solution, EDTA-Na_2_ solution and 30% H_2_O_2_ solution were added sequentially to determine the results. Each sample had three biological replicates and the results were averaged.

Glutathione reductase (GR) activity was determined by glutathione reductase assay kit (Cat no., S0055) from Beyotime Biotechnology, China. The specific steps were followed by the instructions. Each sample had three biological replicates and the results were averaged.

The dehydroascorbate reductase (DHAR) and monodehydroascorbate reductase (MDHAR) activities were determined by the method of Arrigoni with slight modifications [38]. The seeds (0.05 g) were homogenized with 1 ml extraction solution on ice and centrifuged at 10,000 rpm/min for 30 min at 4℃. Then, the supernatant was taken and sequentiall added DHAR and MDHAR reaction solution to determine the results. Each sample had three biological replicates and the results were averaged.

### RNA sequencing

Total RNA was extracted using the ethanol precipitation method and CTAB-PBIOZOL. After successful extraction, RNA was dissolved by adding 50 µL of DEPC treated water. Subsequently, total RNA was identified and quantified using a Qubit fluorescence quantifier and a Qsep400 high-throughput biofragment analyzer. Utilizing the structural characteristics of most mRNA in eukaryotes carrying poly A tails, a cDNA library was constructed by enriching mRNA with poly A tails using Oligo (dT) magnetic beads. The cDNA libraries were sequenced on the Illumina sequencing platform by Metware Biotechnology Co., Ltd. (Wuhan, China).

The raw data were filtered using fastp to obtain high-quality reads after filtering [39]. All subsequent analyses were based on clean reads. Transcriptome assembly of clean reads was performed using Trinity, and the assembled transcripts were clustered and de-redundant using Corset (https://github.com/trinityrnaseq/trinityrnaseq) [40]. Transdecoder (https://github.com/Transdecoder/) was used to perform CDS prediction of Trinity-assembled transcripts to obtain the corresponding amino acid sequences. The expression level of transcripts was calculated using RSEM software, and then the FPKM of each transcript was calculated to estimate the gene expression level based on the transcript length [41]. In order to identify genes related to seed viability of *E. mollis*, differential expression analysis of different subgroups of samples was performed using DESeq2 [42–43]. The differential gene screening criteria were as follows: log2Fold Change ≥ 1 and FDR < 0.05. Furthermore, the differential genes underwent functional annotation and enrichment analysis [44]. Transcription factor prediction was performed using iTAK.

### Metabolic analysis

Using vacuum freeze-drying technology, place the biological samples in a lyophilizer (Scientz-100 F), then grind (30 Hz, 1.5 min) the samples to powder form by using a grinder (MM 400, Retsch). Then weigh 50 mg of sample powder using an electronic balance (MS105DΜ) and add 1200 µL of -20 °C pre-cooled 70% methanolic aqueous internal standard extract (less than 50 mg added at the rate of 1200 µL extractant per 50 mg sample). Vortex once every 30 min for 30 s, for a total of 6 times. After centrifugation (rotation speed 12000 rpm, 3 min), the supernatant was aspirated, and the sample was filtered through a microporous membrane (0.22 μm pore size) and stored in the injection vial for UPLC-MS/MS analysis.

The sample extracts were analyzed using an UPLC-ESI-MS/MS system (UPLC, ExionLC™ AD, https://sciex.com.cn/) and Tandem mass spectrometry system (https://sciex.com.cn/). The analytical conditions were as follows, UPLC: column, Agilent SB-C18 (1.8 μm, 2.1 mm * 100 mm). The mobile phase was consisted of solvent A, pure water with 0.1% formic acid, and solvent B, acetonitrile with 0.1% formic acid. Sample measurements were performed with a gradient program that employed the starting conditions of 95% A, 5% B. Within 9 min, a linear gradient to 5% A, 95% B was programmed, and a composition of 5% A, 95% B was kept for 1 min. Subsequently, a composition of 95% A, 5.0% B was adjusted within 1.1 min and kept for 2.9 min. The flow velocity was set as 0.35 mL per minute; The column oven was set to 40 °C. The injection volume was 2 µL.

The electrospray ionization (ESI) source operation parameters were as follows: source temperature 550 °C; ion spray voltage (IS) 5500 V (positive ion mode)/-4500 V (negative ion mode); ion source gas I (GSI), gas II (GSII), curtain gas (CUR) were set at 50, 60, and 25 psi, respectively. The collision-activated dissociation (CAD) was high. QQQ scans were acquired as MRM experiments with collision gas (nitrogen) set to medium. DP (declustering potential) and CE (collision energy) for individual MRM transitions was done with further DP and CE optimization. A specific set of MRM transitions were monitored for each period according to the metabolites eluted within this period.

Metabolite quantification was analyzed in a multiple reaction monitoring (MRM) mode using triple quadrupole mass spectrometry [45]. Mass spectrometry data were processed using the software Analyst 1.6.3. Differences between sample metabolites were considered significant when the variable importance projection (VIP) > 1 and fold change ≥ 2 and fold change ≤ 0.5. The R software modeling package was used for grouped orthogonal partial least squares discriminant analysis (OPLS-DA) and kyoto encyclopedia of genes and genomes (KEGG) analysis to gain insights into the metabolic pathways associated with seed viability in *E. mollis* [46–47]. Three biological replicates were used for each sample.

## Results

### Viability analysis of *E. mollis* seeds

**Fig. 1:**
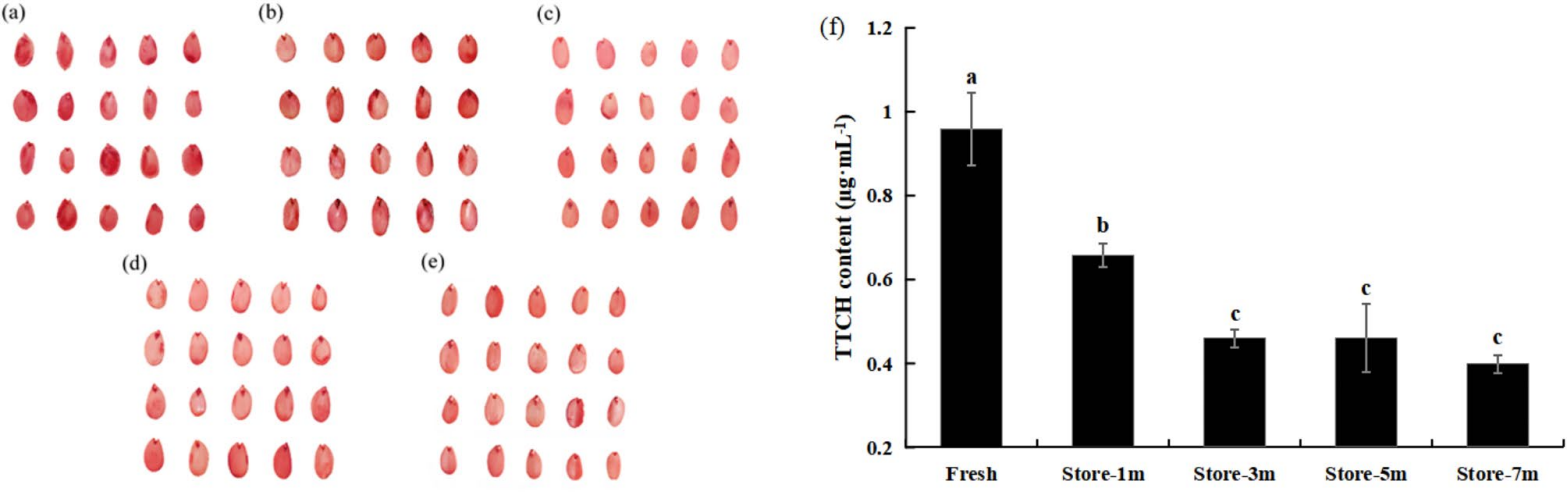
Changes in viability of *E. mollis* seeds after four storage times at room temperature. Notes: TTC staining chart with red indicating viable tissues (**a**) Fresh seeds. (**b**) seeds stored for 1 month. (**c**) seeds stored for 3 months. (**d**) seeds stored for 5 months. (**e**) seeds stored for 7 months. (**f**) TTCH quantitative analysis of the five treatments. Different lowercase letters represent significant differences between treatments (*p* ≤ 0.05)

In the natural state, after 1 month of storage at room temperature, the viability of the seeds of *E. mollis* began to decline substantially. After 3 months of storage, viability again decreased significantly. After 5 and 7 months of storage, the trend of decreasing viability from that of 3 months of storage was not significant (Fig. 1).

### Physiological indexes analysis of seed viability in *E. mollis*

**Fig. 2:**
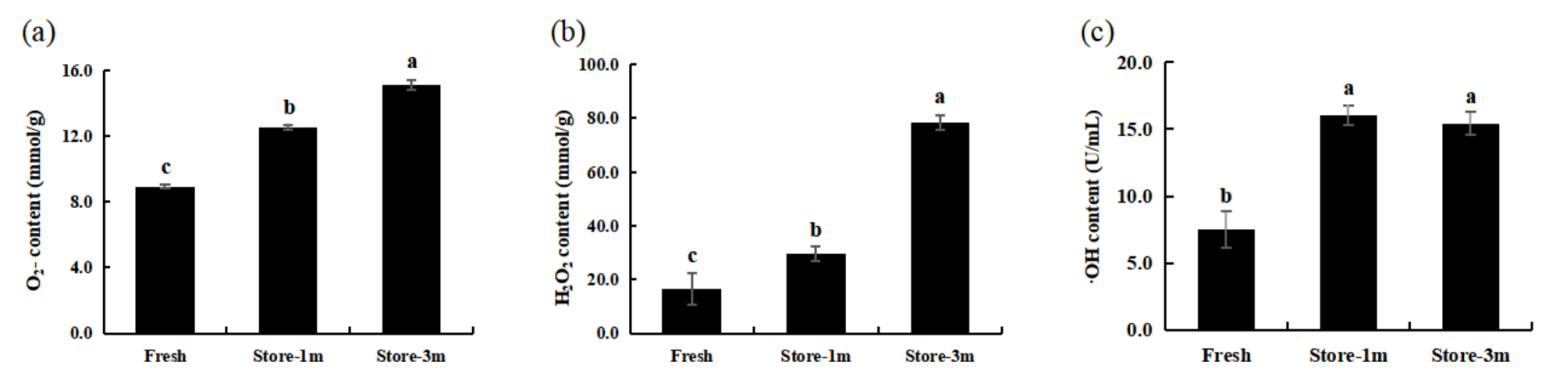
Changes in the content of the main components of ROS in the treatment groups of fresh and stored *E. mollis* seeds. (**a**) O_2_^−^ content. (**b**) H_2_O_2_ content. (**c**) ·OH content. Notes: Different lowercase letters represent significant differences between treatments (*p* ≤ 0.05)

After 1 and 3 months, O_2_^−^ and H_2_O_2_ contents were significantly higher than in fresh seeds, and the difference was also significant between 1 and 3 months. While ·OH content was significantly higher than that of fresh seeds after 1 month and 3 months, the difference was not significant between 1 month and 3 months (Fig. 2).

**Fig. 3:**
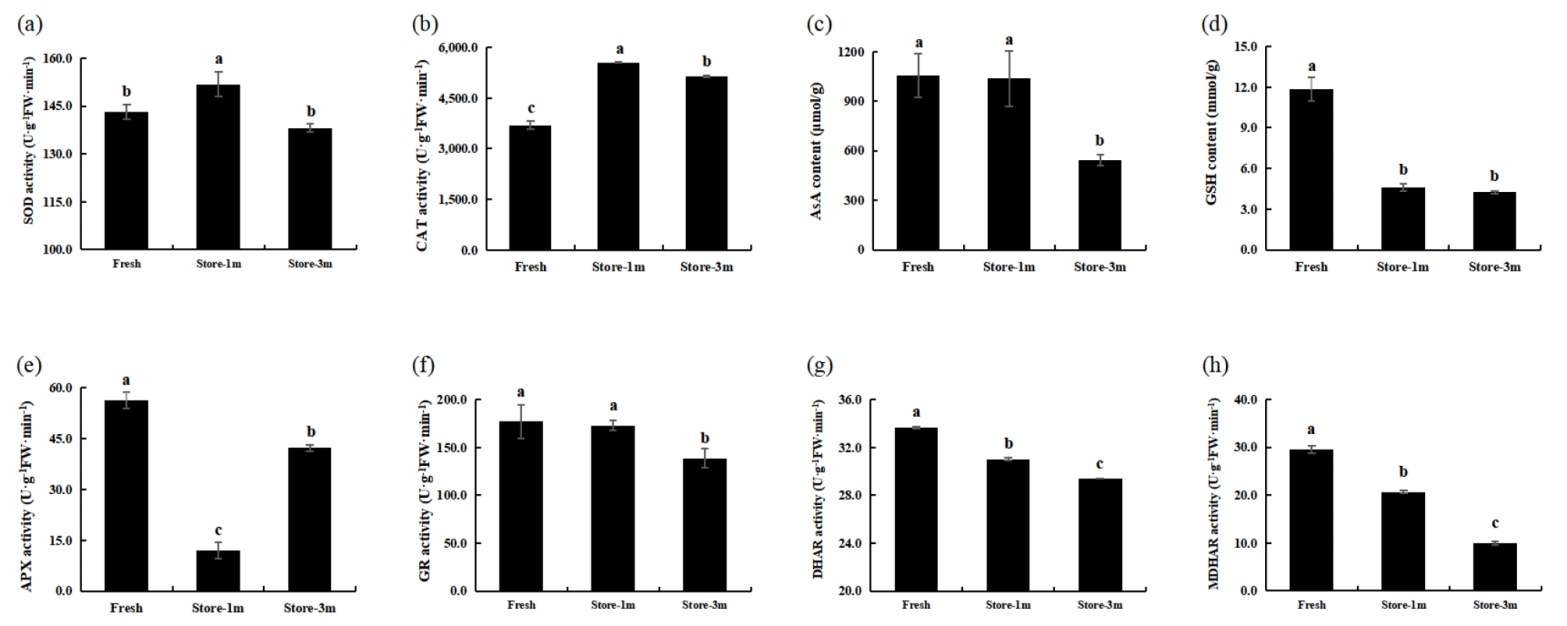
Changes in indicators related to antioxidant defense system of different storage time treatment groups of *E. mollis* seeds. (**a**) SOD activity. (**b**) CAT activity. (**c**) AsA content. (**d**) GSH content. (**e**) APX activity. (**f**) GR activity. (**g**) DHAR activity. (**h**) MDHAR activity. Notes: Different lowercase letters represent significant differences between treatments (*p* ≤ 0.05)

Compared with fresh seeds, the activities of DHAR and MDHAR decreased continuously with increased storage time. After 1 month, the activities of SOD and CAT were significantly higher than that of fresh seeds, and both significant decreased after 3 months of storage, but CAT activity was still significantly higher than fresh seeds. The content of GSH decreased significantly after 1 month of storage, and there was no significant change between 3 months and 1 month of storage. APX activity decreased significantly after 1 month of storage and increased significantly after 3 months of storage, but its activity was still significantly lower than that of fresh seeds. In addition, AsA content and GR activity were significantly decreased only after 3 months of storage (Fig. 3).

### Transcriptomic analysis of seed viability in *E. mollis*

To further investigate the molecular mechanisms underlying the decline in seed viability of *E. mollis*, we conducted transcriptome sequencing on fresh seeds, seeds stored for 1 month, and seeds stored for 3 months. A total of 483,288,434 Clean Reads were obtained, in which the percentage of Q30 bases was 93% and above, and the percentage of GC was 43% and above (Table S1), indicating that the transcriptome data were reliable and could be analyzed in the next step.

**Fig. 4:**
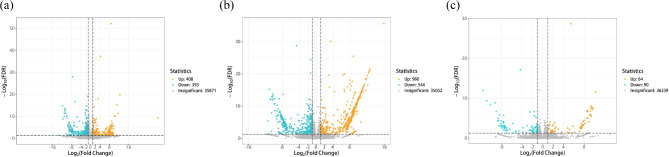
EDGs volcano plots of different preservation time treatment groups of *E. mollis* seeds. Notes: The horizontal axis represents changes in gene expression multiples; The vertical axis represents the significance level of DEGs. (**a**) Stored for 1 month vs. fresh seeds. (**b**) Stored for 3 months vs. fresh seeds. (**c**) Stored for 3 months vs. stored for 1 month. Blue indicates decreases and orange indicates increases in expression

**Fig. 5:**
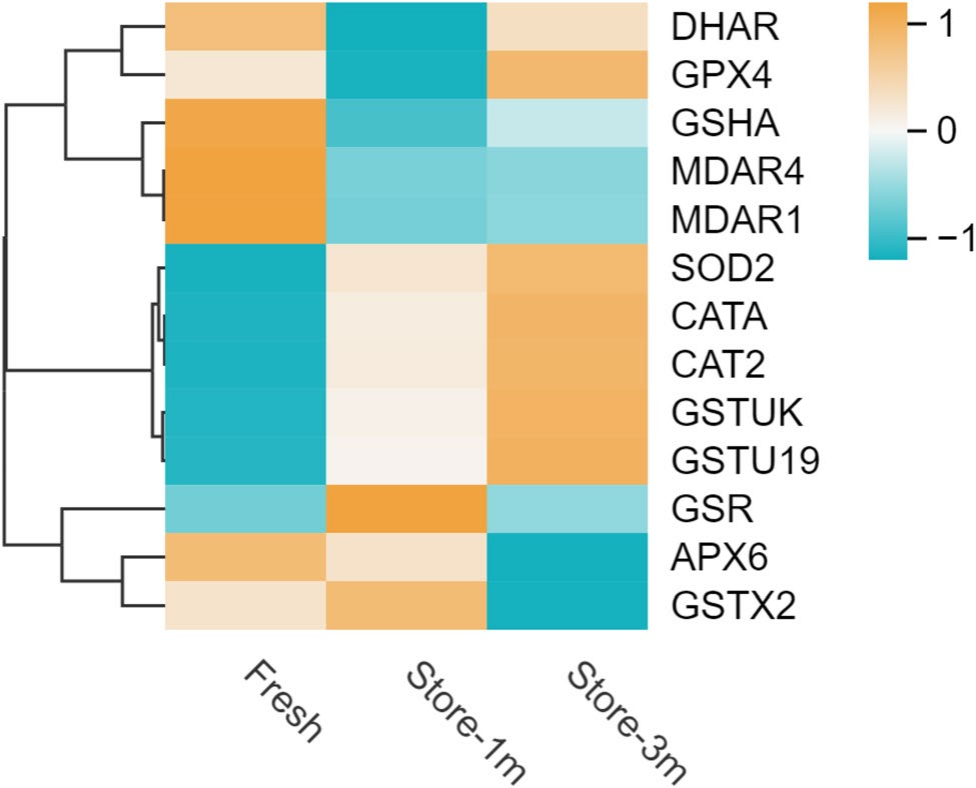
Heatmap of DEGs associated with the antioxidant defense system of *E. mollis* seeds. Notes: The horizontal coordinate represents the sample name, and the vertical coordinate represents the differential gene and hierarchical clustering results. Orange indicates high expression and blue indicates low expression

A total of 76,429 genes were identified in the transcriptome gene functional annotation of *E. mollis* seeds, which were stored for different lengths of time. In total, 61,257 genes were annotated in the NR database, and 47,939 genes were annotated in the KEGG database. A total of 61,801 genes were successfully annotated by all databases, accounting for 80.86% of the total number of genes (Table 1). A total of 801 significantly altered differentially expressed genes (DEGs) were identified in fresh seeds and seeds stored for 1 month, of which 408 were upregulated and 393 were downregulated (Fig. 4a). 1,524 significantly altered DEGs were identified between seeds stored for 3 months and fresh seeds, of which 980 were upregulated and 544 were downregulated (Fig. 4b). And 154 significantly changed DEGs were identified between seeds stored for 3 months and seeds stored for 1 month, with 64 upregulated and 90 downregulated (Fig. 4c). Differential genes were further analyzed and screened to obtain DEGs related to antioxidant defense system based on KEGG database annotation (Fig. 5). A total of 13 significantly changed DEGs were detected, including 2 *CAT*, 1 *SOD*, 1 *GR*, 1 *GSH*, 3 *GST*, 2 *MDAR*, 1 *APX*, 1 *DHAR* and 1 *GPX*. After 1 month of storage, the gene expressions of catalase (*CAT*2, *CATA*), superoxide dismutase (*SOD*2), and glutathione metabolism cycle (*GSR*, *GSTU*19, *GSTUK*, *GSTX2*) in the seeds of *E. mollis* were significantly upregulated, but the genes of glutathione metabolism cycle (*MDAR*1, *MDAR*4, *GSHA*, *DHAR*, *GPX4*), and ascorbic peroxidase (*APX*6) were significantly downregulated. In addition, after 3 months of storage, the expression levels of glutathione metabolism cycle (*GSHA*, *DHAR*) increased, while the expression levels of glutathione metabolism cycle (*GSR*) decreased. It is worth noting that the expression levels of *CAT*, *MDHAR*, *GSH* and *GR* genes were consistent with the results of the physiological indicators mentioned above, except for *DHAR*.

**Table 1:** Transcriptome data in the seven databases and success rate statistics of the gene annotation

Database	Number of Genes	Percentage (%)
KEGG	47,939	62.72
NR	61,257	80.15
SwissProt	46,924	61.40
TrEMBL	61,190	80.06
KOG	39,618	51.84
GO	53,246	69.67
Pfam	43,552	56.98
Annotated in at least one Database	61,801	80.86
Total Unigenes	76,429	100

Abbreviations: KEGG, Kyoto Encyclopedia of Genes and Genomes; NR, non-redundant protein sequences; SwissProt, Swissprot Protein Sequence; TrEMBL, Translation of EMBL; KOG, euKaryotic Ortholog Groups; GO, Gene Ontology; Pfam, Protein family

**Fig. 6:**
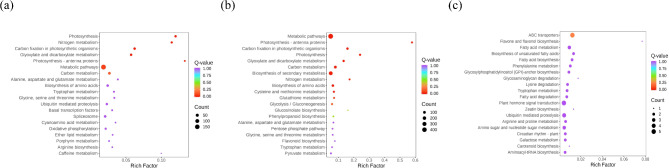
KEGG enrichment analysis of DEGs in the groups treated with different preservation times of *E. mollis* seeds. (**a**) Stored for 1 month vs. fresh seeds. (**b**) Stored for 3 months vs. fresh seeds. (**c**) Stored for 3 months vs. stored for 1 month. Notes: The ordinate indicates the KEGG pathway. The horizontal coordinate represents the rich factor. The larger the dot, the greater the number of pathway enriched differential genes. The redder the dot, the more significant the enrichment

The genes were annotated into the KEGG database, and the 20 pathways with the most significant enrichment were selected for presentation, and drawn in the enrichment scatter plot (Fig. 6). The pathways related to seed viability in *E. mollis* include metabolic pathways, carbon fixation in photosynthetic organisms, glyoxylate and dicarboxylate metabolism, nitrogen metabolism, alanine, aspartate and glutamate metabolism, tryptophan metabolism and plant hormone signal transduction.

### Metabolomic analysis of seed viability in *E. mollis*

**Fig. 7:**
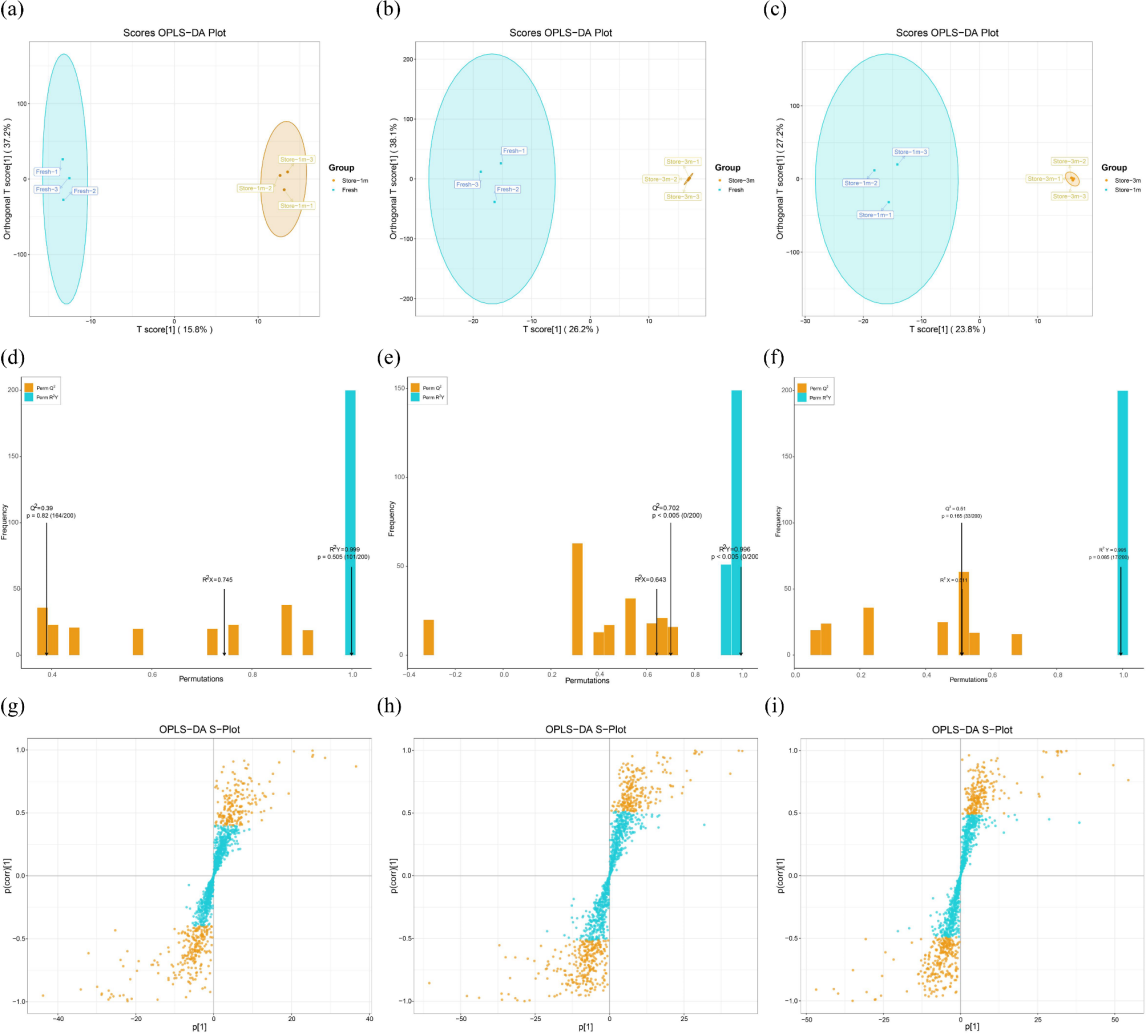
DAMs OPLS-DA analysis of different preservation time treatment groups of *E. mollis* seeds. (**a**) OPLS-DA scores analysis of stored for 1 month vs. fresh seeds. (**b**) OPLS-DA scores analysis of stored for 3 months vs. fresh seeds. (**c**) OPLS-DA scores analysis of stored for 3 months vs. stored for 1 month. (**d**) OPLS-DA validation of stored for 1 month vs. fresh seeds. (**e**) OPLS-DA validation of stored for 3 months vs. fresh seeds. (**f**) OPLS-DA validation of stored for 3 months vs. stored for 1 month. (**g**) OPLS-DA S-plot of stored for 1 month vs. fresh seeds. (**h**) OPLS-DA S-plot of stored for 3 months vs. fresh seeds. (**i**) OPLS-DA S-plot of stored for 3 months vs. stored for 1 month

**Fig. 8:**
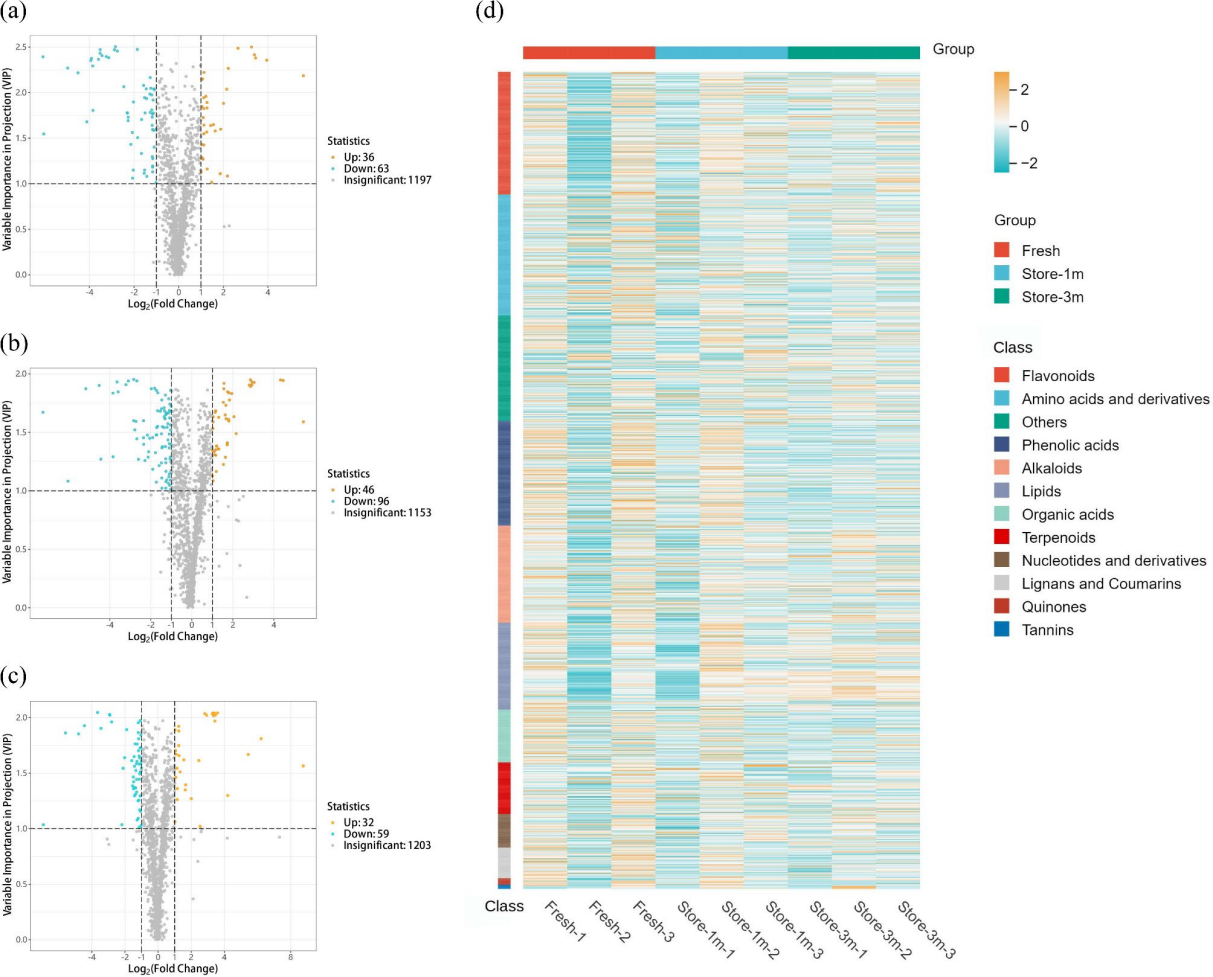
DAMs analysis of the groups treated with different preservation times of *E. mollis* seeds. Notes: (**a**) Volcano plot of DAMs between stored for 1 month and fresh seeds. (**b**) Volcano plot of DAMs between stored for 3 months and fresh seeds. (**c**) Volcano plot of DAMs between stored for 3 months and stored for 1 month. (**d**) Clustering heatmap of DAMs

Based on the OPLS-DA model (Fig. 7), we selected metabolites with VIP > 1, fold change ≥ 2, and fold change ≤ 0.5. A total of 1,298 metabolites were obtained from fresh seeds, seeds stored for 1 month, and seeds stored for 3 months. Among them, A total of 99 significantly altered differentially altered metabolites (DAMs) were identified in fresh seeds and seeds stored for 1 month, with 36 upregulated and 63 downregulated (Fig. 8a). A total of 142 significant DAMs were identified between seeds stored for 3 months and fresh seeds, of which 46 were upregulated and 96 were downregulated (Fig. 8b). A total of 91 significant DAMs were identified between seeds stored for 3 months and stored for 1 month, of which 32 were upregulated and 59 were downregulated (Fig. 8c). Moreover, the main types of DAMs were flavonoids, amino acids and derivatives, and phenolic acids (Fig. 8d).

**Fig. 9:**
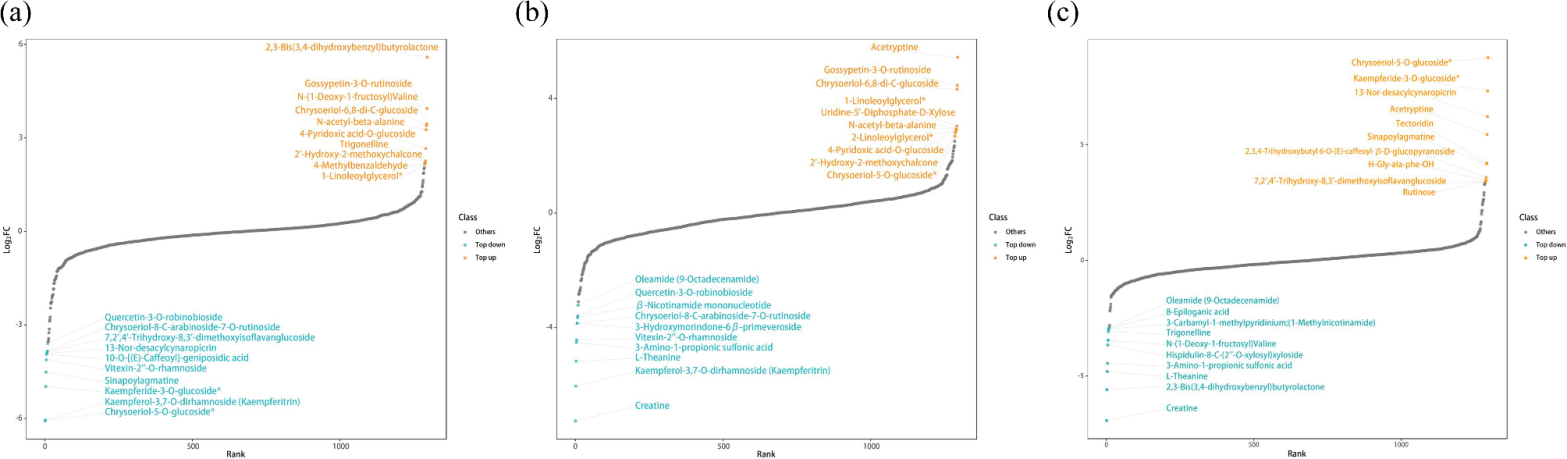
DAMs dynamic distribution plot of the groups treated with different preservation times of *E. mollis* seeds. Notes: The horizontal axis represents the cumulative number of substances arranged in ascending order according to the difference multiple. The vertical axis represents the logarithmic value of the difference multiple with a base of 2. (**a**) Stored for 1 month vs. fresh seeds. (**b**) Stored for 3 months vs. fresh seeds. (**c**) Stored for 3 months vs. stored for 1 month

Concurrently, the detected metabolites were analyzed qualitatively and quantitatively. Based on the calculated fold change (FC) values arranged from small to large, we drew a dynamic distribution plot of metabolite content differences (Fig. 9), and labelled the top 10 metabolites which upregulated and downregulated. The results showed that some key metabolic substances, such as quercetin-3-O-robinobioside, chrysoeriol-8-C-arabinoside-7-O-rutinoside, vitexin-2”-O-rhamnoside, kaempferol-3,7-O-dirhamnoside (kaempferitrin), oleamide (9-Octadecenamide), 3-Amino-1-propionic sulfonic acid, l-theanine, and creatine were significantly reduced in expression with prolonged storage time. While, the expression of gossypetin-3-O-rutinoside, chrysoeriol-6,8-di-C-glucoside, n-acetyl-beta-alanine, 4-pyridoxic acid-O-glucoside, 2’-hydroxy-2-methoxychalcone, 1-linoleoylglycerol*, and acetryptine were increased.

**Fig. 10:**
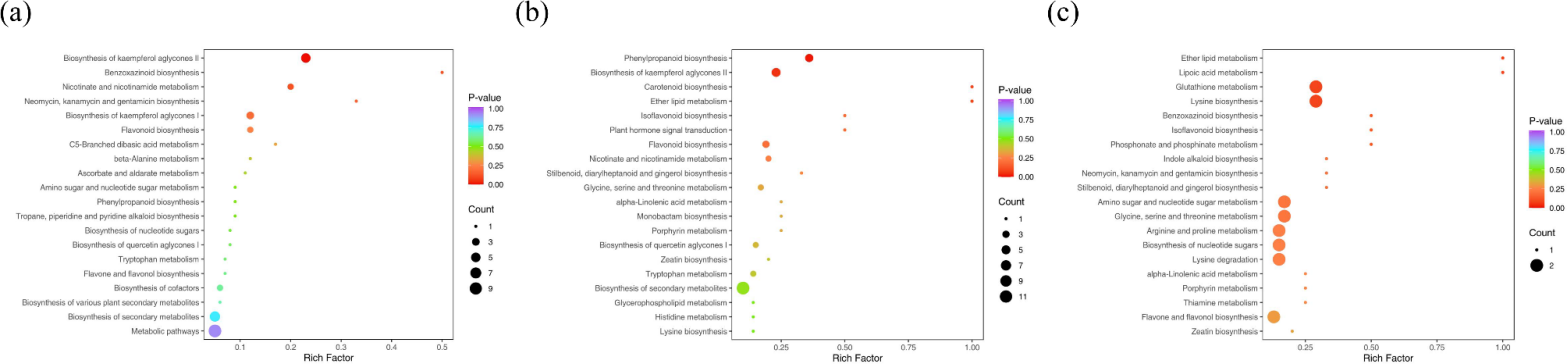
DAMs Pathway enrichment analysis. (**a**) Stored for 1 month vs. fresh seeds. (**b**) Stored for 3 months vs. fresh seeds. (**c**) Stored for 3 months vs. stored for 1 month. Notes: The ordinate indicates the KEGG pathway. The horizontal coordinate represents the rich factor. The larger the dot, the greater the number of pathway enriched differential genes. The redder the dot, the more significant the enrichment

Classify and display the annotation results of significantly DAMs KEGG and metmap (The KEGG pathway classification information was obtained from the KEGG database). According to the classification results of KEGG pathways, most of the DAMs compared between seeds stored for 1 month and fresh seeds were enriched in the metabolic pathways, biosynthesis of secondary metabolites, biosynthesis of cofactors, flavonoid biosynthesis, nicotinate, and nicotinamide metabolism pathways (Fig. 10a). The comparison between seeds stored for 3 months and fresh seeds were mainly enriched in the metabolic pathways, biosynthesis of secondary metabolites, phenolpropanoid biosynthesis, biosynthesis of amino acids, and flavonoid biosynthesis pathways (Fig. 10b). The comparison between seeds stored for 3 months and stored for 1 month were mainly enriched in flavone and flavonol biosynthesis, glutathione metabolism, and lysine biosynthesis (Fig. 10c).

### Transcriptomic and metabolomic analysis of *E. mollis* seed viability

**Fig. 11:**
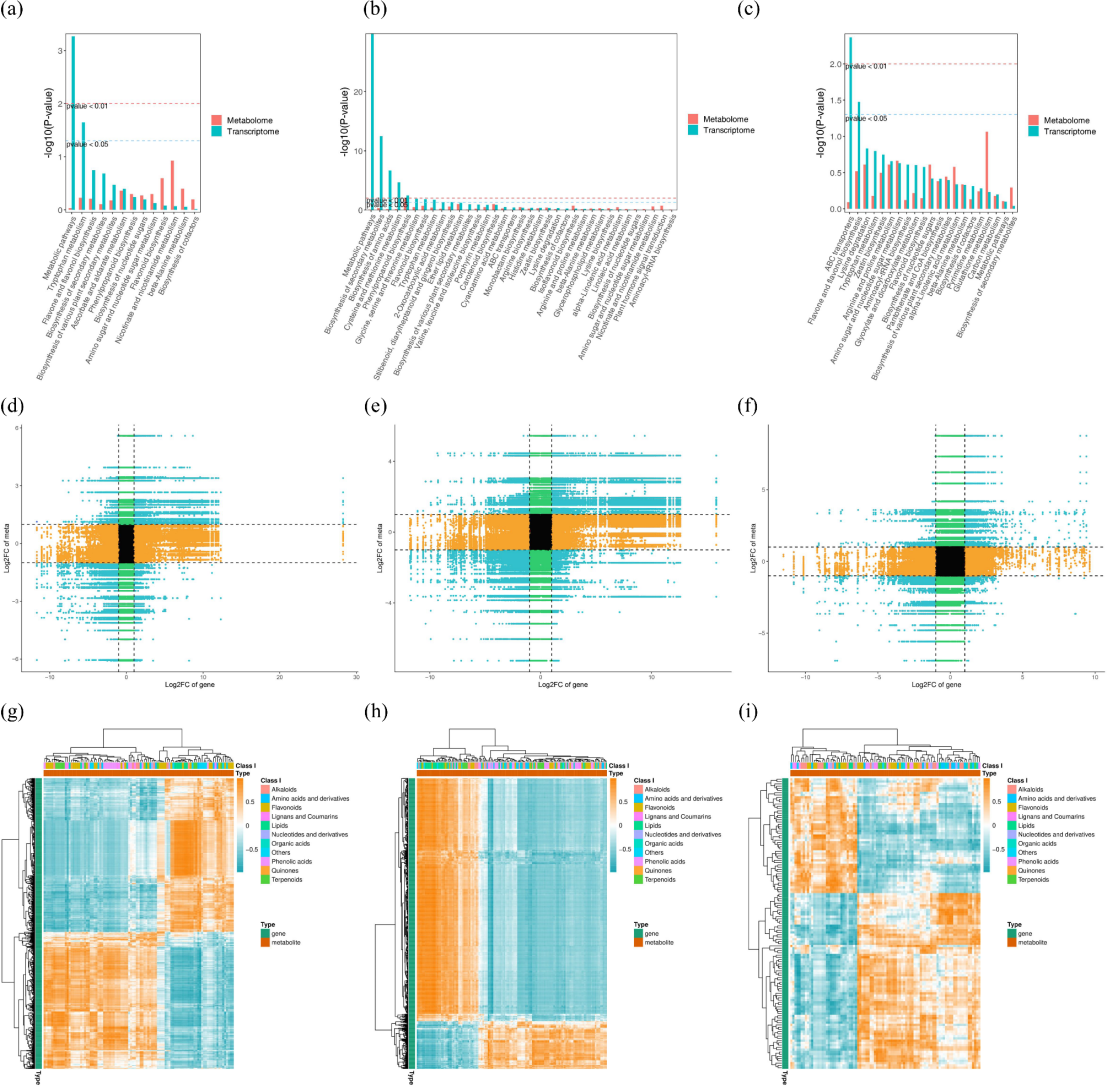
Combined analysis of DEGs and DAMs of the groups treated with different preservation times of *E. mollis* seeds. (**a**) Bar graph of KEGG enrichment analysis of stored for 1 month vs. fresh seeds. (**b**) Bar graph of KEGG enrichment analysis of stored for 3 months vs. fresh seeds. (**c**) Bar graph of KEGG enrichment analysis of stored for 3 months vs. stored for 1 month. (**d**) Nine-quadrant correlation analysis of stored for 1 month vs. fresh seeds. (**e**) Nine-quadrant correlation analysis of stored for 3 months vs. fresh seeds. (**f**) Nine-quadrant correlation analysis of stored for 3 months vs. stored for 1 month. (**g**) Clustering heatmap of stored for 1 month vs. fresh seeds. (**h**) Clustering heatmap of stored for 3 months vs. fresh seeds. (**i**) Clustering heatmap of stored for 3 months vs. stored for 1 month

DEGs and DAMs analysis were conducted for seeds stored for 1 month and 3 months and fresh seeds. The results of KEGG pathway enriched by the two omics showed that 13 pathways were enriched between seeds stored for 1 month and fresh seeds (Table S2), with significant enrichment in metabolic pathways and tryptophan metabolism pathways (Fig. 11a). While 35 pathways were found to be significantly enriched between seeds stored for 3 months and fresh seeds (Table S3). These pathways were primarily associated with metabolic processes, including metabolic pathways, biosynthesis of secondary metabolites, biosynthesis of amino acids, cysteine and methionine metabolism, phenolpropanoid biosynthesis, glycine, serine and threonine metabolism, flavonoid biosynthesis, and tryptophan metabolism (Fig. 11b). Also, 21 pathways were found to be significantly enriched between seeds stored for 3 months and 1 month (Table S4), with significant enrichment in ABC transporters, and flavone and flavonol biosynthesis (Fig. 11c).

Based on the Pearson correlation coefficient, we selected the correlation parts with an absolute value greater than 0.8 and a p value less than 0.05. Then displayed the differences of multiples of genes and metabolites corresponding to these correlation parts through a 9-quadrant graph (Fig. 11d, e, f). The differential expression patterns of genes and metabolites distributed in the third and seventh quadrants were consistent, and there was a positive correlation between them. The change of metabolites may be the result of positive gene regulation. Among them, a total of 23,713 genes were positively correlated with 107 metabolites in fresh seeds and seeds stored for 1 month. While, 38,326 genes were positively correlated with 176 metabolites in seeds stored for 3 months and fresh seeds. And 6,419 genes were positively correlated with 105 metabolites in seeds stored for 3 months and stored for 1 month. The expression patterns of genes and metabolites distributed in the first and ninth quadrants were opposite, and there was a non-uniform regulatory trend between them. The expression of metabolites may be negatively regulated by genes. Among them, a total of 5,006 genes were identified as negatively correlated with 97 metabolites in fresh seeds and seeds stored for 1 month. While, a total of 9,295 genes were negatively correlated with 169 metabolites in seeds stored for 3 months and fresh seeds. And a total of 2,250 genes were negatively correlated with 103 metabolites in seeds stored for 3 months and stored for 1 month. Subsequently, we selected all correlation calculation results of DEGs and DAMs, and drew correlation clustering heatmaps. The results showed that a large number of DEGs and DAMs were correlated, among which the DEGs and DAMs of fresh seeds and stored for 1 month seeds were mainly flavonoids, lipids, and phenolic acids (Fig. 11g). Lipids and flavonoids were the main DEGs and DAMs between fresh seeds and stored for 3 months (Fig. 11h). Moreover, DEGs and DAMs of seeds stored for 3 months and 1 month were dominated by flavonoids and phenolic acids (Fig. 11i).

### DEGs and dams of flavonoid biosynthesis-related May affect *E. mollis* seed viability

**Fig. 12:**
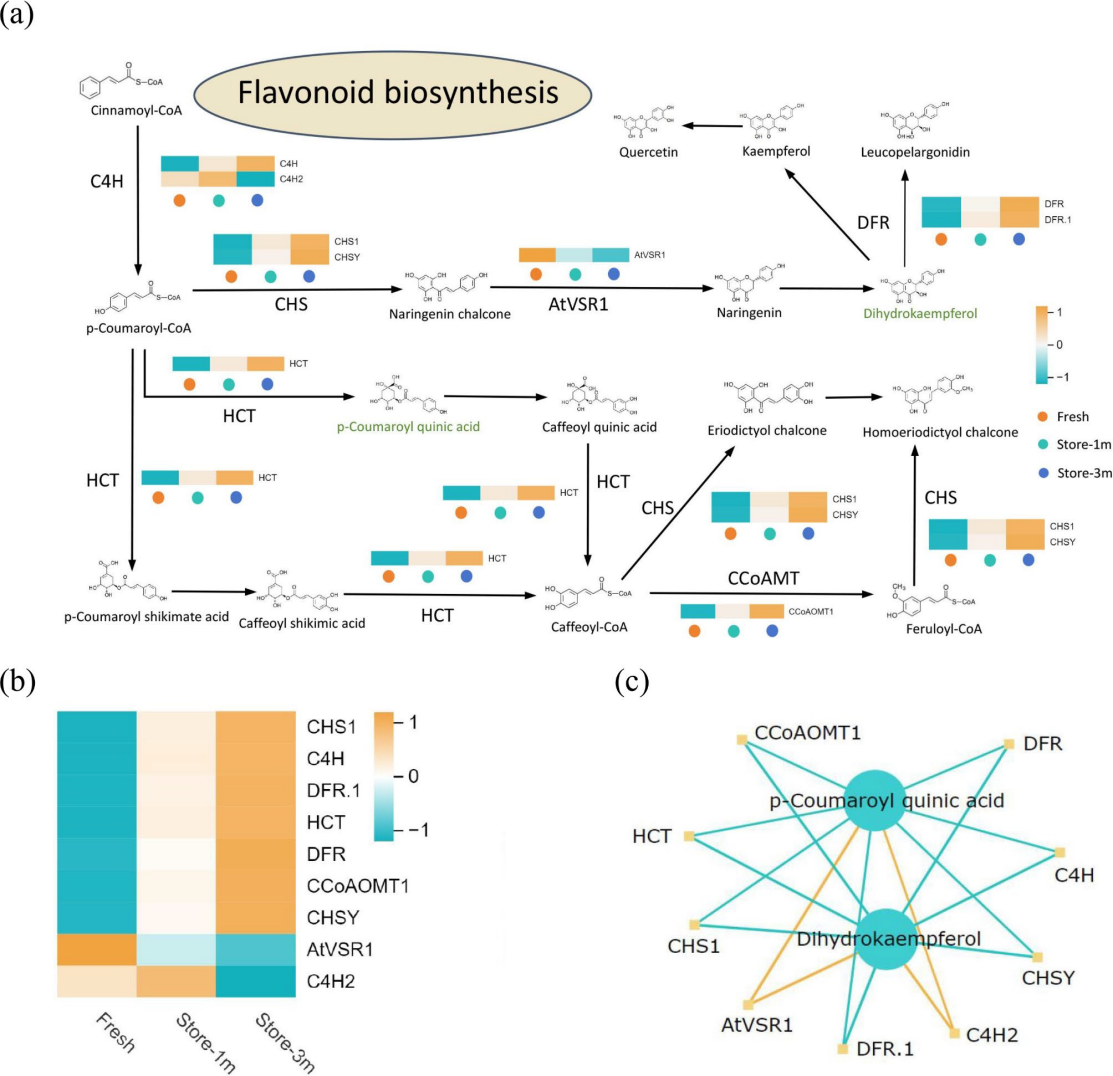
(**a**) Expression profiling of key gene families in the flavonoid biosynthesis. 6 critical structural gene families were identified: *CHS* (chalcone synthase), *C4H* (trans-cinnamate), *HCT* (shikimate), *DFR* (bifunctional dihydroflavonol), *AtVSR1* (chalcone isomerase), and *CCoAOMT* (caffeoyl-CoA) (Green represents downregulation). (**b**) DEGs related to flavonoid biosynthesis. (**c**) Correlation network between DEGs and DAMs in flavonoid biosynthesis (The orange line indicates a positive correlation and the blue line indicates a negative correlation)

Enrichment analysis showed that DEGs and DAMs between seeds stored for 1 month and 3 months and fresh seeds were significantly enriched in the flavonoid biosynthesis pathway. Consequently, we reconstructed the flavonoid biosynthesis pathway with the objective of elucidating the role of this pathway in the decline of seed viability in *E. mollis* (Fig. 12a). A total of six key genes were detected: *CHS* (chalcone synthase), *C4H* (trans-cinnamate), *HCT* (shikimate), *DFR* (bifunctional dihydroflavonol), *AtVSR*1 (chalcone isomerase), and *CCoAOMT* (caffeoyl-CoA). Among them, *CHS*, *HCT*, *CCoAOMT*, and *DFR* showed similar expression patterns. The correlation between key genes and metabolites are represented through a network graph, and key DEGs and DAMs with Pearson correlation coefficient absolute value greater than 0.8 and p value less than 0.05 which were selected from each pathway and plotted as correlation results (Fig. 12b, c). Two key genes were positively correlated, while seven were negatively correlated in the flavonoid biosynthesis network. Dihydrokaempferol and p-Coumaroyl quinic acid were key metabolites that linked key genes in the flavonoid biosynthesis pathway.

### Tryptophan metabolism-related genes May affect *E. mollis* seed viability

**Fig. 13:**
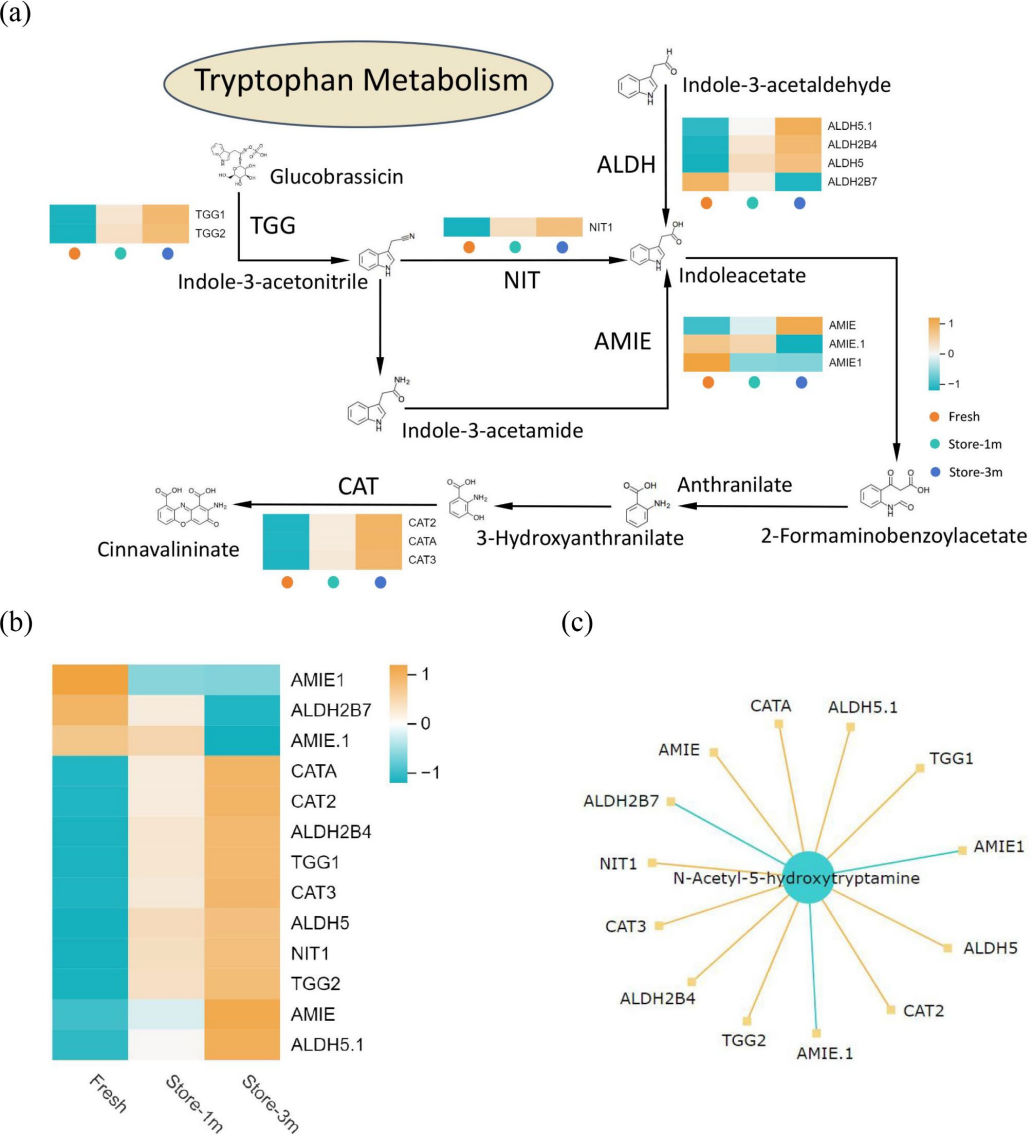
(**a**) Expression profiling of key gene families in the tryptophan metabolism. 5 critical structural gene families were identified: *ALDH* (aldehyde dehydrogenase), *NIT* (nitrilase), *AMIE* (amidase), *TGG* (myrosinase), and *CAT* (catalase). (**b**) DEGs related to tryptophan metabolism. (**c**) Correlation network between DEGs and DAMs in tryptophan metabolis (The orange line indicates a positive correlation and the blue line indicates a negative correlation)

Conjoint Analysis of DEGs and DAMs showed that the transcriptome and metabolomics of *E. mollis* seeds were enriched in the Tryptophan pathway. Therefore, we furthered our analysis of the role of this pathway in the decline of seed viability in *E. mollis* (Fig. 13a). Five key genes were detected: *ALDH* (aldehyde dehydrogenase), *NIT* (nitrilase), *AMIE* (amidase), *TGG* (myrosinase), and *CAT* (catalase). In this pathway, similar expression patterns were observed for *TGG*, *NIT*, and *CAT* genes. The correlation between key genes and metabolites were represented through a network graph, and key DEGs and DAMs with Pearson correlation coefficient absolute value greater than 0.8 and p value less than 0.05 were selected from each pathway and plotted as correlation results (Fig. 13b, c). Ten key genes were positively correlated, while three were negatively correlated in the tryptophan metabolism network. And N-Acetyl-5-hydroxytryptamine was a key metabolite that linked key genes in the tryptophan metabolism pathway.

### Transcription factors (TFs) associated with loss of seed viability in *E. mollis*

**Fig. 14:**
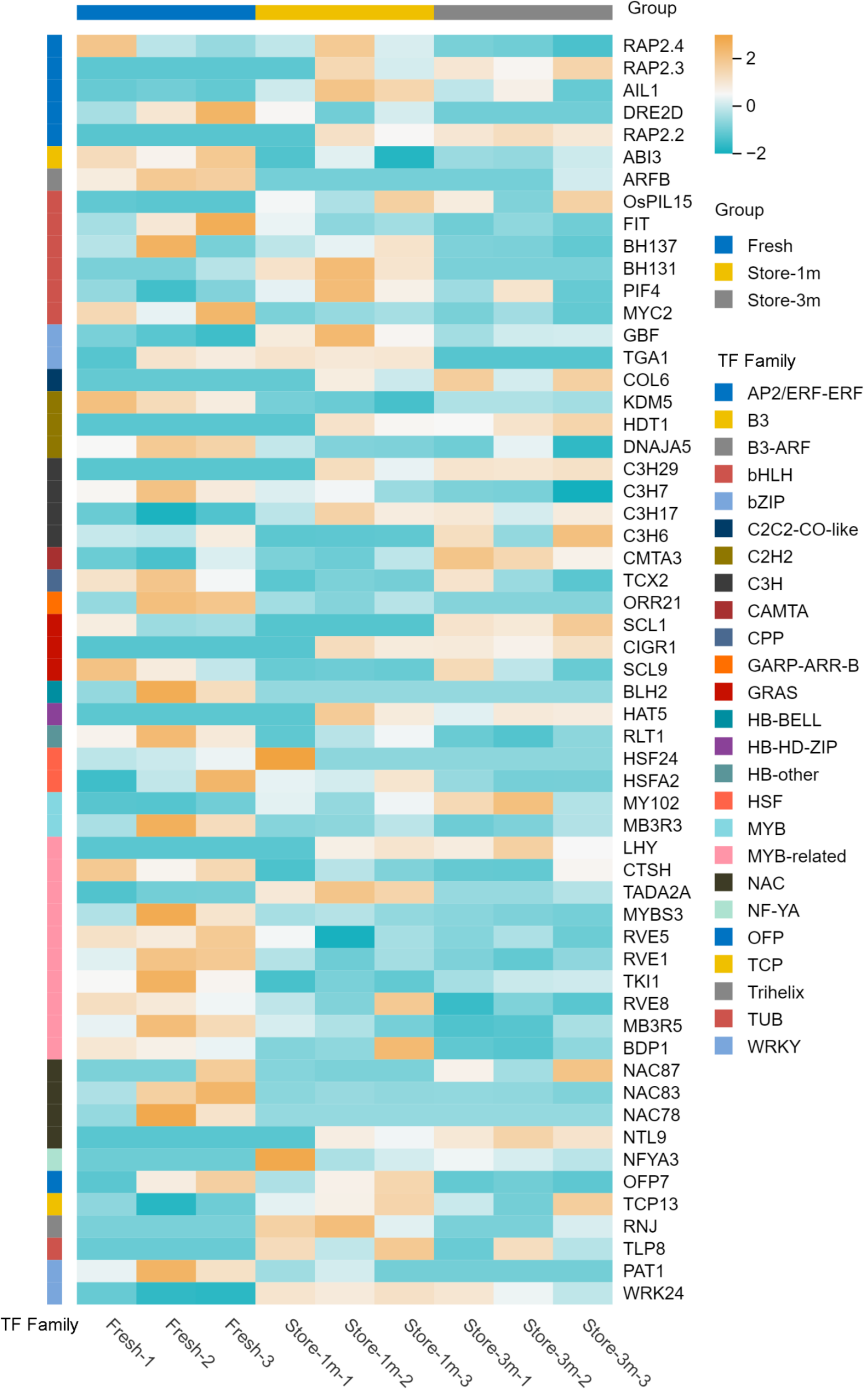
Expression profiling of TFs regulating seed viability in *E. mollis*

We used iTAK 1.7a to predict plant transcription factors and identified 25 TFs involved in the regulation of seed viability changes in *E. mollis*. Most TF families were down-regulated during the process of seed viability decline. Among them, we found that the top five TF families with the most members were *MYB-related* (10 members: *LHY*, *CTSH*, *TADA2A*, *MYBS3*, *RVE5*, *RVE1*, *TKI1*, *RVE8*, *MB3R5*, *BDP1*), *bHLH* (6 members: *OsPIL15*, *FIT*, *BH137*, *BH131*, *PIF4*, *MYC2*), *AP2/ERF-ERF* (5 members: *RAP2.4*, *RAP2.3*, *AIL1*, *DRE2D*, *RAP2.2*), *C3H* (4 members: *C3H29*, *C3H7*, *C3H17*, *C3H6*) and *NAC* (4 members: *NAC87*, *NAC83*, *NAC78*, *NTL9*). In addition, the *bZIP*, *WRKY*, *MYB*, and *B3* transcription factors play an important role in responding to abiotic stress in plants (Fig. 14).

## Discussion

The phenomenon of seed viability decline is inevitable during seed storage, and low viability seeds can significantly affect population densities [48]. Especially in the case of rare and endangered plants, it may result in the endangerment of species, which is very detrimental to the conservation and utilization of germplasm resources [49]. In this study, the seed viability of *E. mollis* exhibited a marked decline after 1 month storage, and after 3 months storage seed viability still decreased significantly. Howevere seed stored for 5 and 7 months declined slowly, and the difference in viability from seed stored for 3 months was not significant (Fig. 1). These observations were consistent with the changes in viability observed in the seeds of *Ephedra sinica*, *Elymus sibiricus*, and *Hibiscus cannabinus* in their natural storage state [50]. It can be observed that viability decline is a common phenomenon during seed storage, and the viability decline of *E. mollis* seeds is particularly severe.

With the development of molecular biology, the omics technology has gradually become a research hotspot. Combining transcriptomics and metabolomics to analyze the genes and metabolites related to seed viability can help us understand the biological process of viability decline and its mechanism more intuitively and effectively [51–53]. This study identified 801 and 1,524 significantly DEGs between fresh seeds and those stored for 1 and 3 months, and 154 significantly changed DEGs were identified between seeds stored for 3 months and seeds stored for 1 month (Fig. 4). The KEGG enrichment analysis revealed that these DEGs were mainly involved in metabolic pathways, carbon fixation in photosynthetic organisms, glyoxylate and dicarboxylate metabolism, nitrogen metabolism, alanine, aspartate and glutamate metabolism, tryptophan metabolism, and plant hormone signal transduction (Fig. 6). Based on the KEGG database annotations, we found that the expression of genes related to antioxidant system *CAT*2, *CATA*, *SOD*2, *GSR*, and *GSTU*19 were significantly upregulated, and *MDAR*1, *MDAR*4, *GSHA*, *DHAR*, and *APX*6 genes were significantly downregulated after stored for 1 month After 3 month storage, the expression of the genes *GSHA* and *DHAR* increased, and the *GSR* gene expression began to be downregulated. As Luo et al. found during seed aging in *Metasequoia glyptostroboides*, the expression of 5 *CAT* genes related to the antioxidant system showed an upregulation trend with increasing storage time. Meanwhile, the expression of *GST* and *GSH* genes in the AsA-GSH antioxidant cycle system was upregulated and the expression of *GR* was downregulated [54]. This may be due to an imbalance in the antioxidant defense system leading to an increase in ROS, which causes oxidative damage to the cells, resulting in a decrease in seed viability.

Antioxidant defense systems have been shown to be closely associated with seed viability [55]. This system consists of several small molecule antioxidants and antioxidant enzymes [56]. Among them, CAT is the most important H_2_O_2_ scavenging enzyme that catalyzes the conversion of H_2_O_2_ to H_2_O and O_2_ to protect cells from oxidative damage [57]. In this study, both *CAT*2 and *CATA* expression were upregulated in seeds stored for 1 month and 3 months, which was consistent with the changing trend at the physiological level (Fig. 5). At the physiological level, we found that CAT activity was higher than in fresh seeds after 1 month and 3 months (Fig. 3). This indicated that seeds enhanced their antioxidant capacity at the early stage of senescence to maintain high seed viability and that CAT plays an important role in delaying seed senescence [58]. Moreover, the AsA-GSH antioxidant cycling system represents a crucial pathway for the scavenging of ROS in plants [59, 60]. It is mainly through the combined action of AsA, GSH, APX, DHAR, MDHAR, and GR with other antioxidant enzyme to remove excess H_2_O_2_ from the cell and harmonize enzyme activity in the cell [61–62]. DHAR is present in the cytoplasm, mitochondria, and chloroplasts, where it catalyzes the reduction of DHA by GSH to produce AsA and GSSG. It is a key enzyme in the AsA-GSH antioxidant cycling system [63]. MDHAR plays a role in the regeneration of MDHA to AsA, as well as in the final accumulation of AsA [64]. GR catalyzes the reduction of GSSG to GSH, thereby maintaining seed viability, sustaining normal plant growth and development, and improving plant stress tolerance [65]. GST can reduce oxidative damage in cells and has positive significance in plant detoxification [66]. In this study, the transcriptome results showed that the expression of *GSTU*19, *GSTUK*, *GSTX2* and *GSR* genes were significantly upregulated, while *MDAR*1, *MDAR*4, *GSHA* and *DHAR* were all downregulated in seeds stored for 1 month (Fig. 5). Similarly, GSH content, DHAR and MDHAR activity were also decreased in physiological results, but there was no significant change in GR (Fig. 3). Meanwhile, after 3 months storage, the expression of *GSHA* and *DHAR* in seeds increased, while the expression of *GSR* began to decrease (Fig. 5). Similarly, physiological results showed decreasing GR activity, but DHAR activity showed the opposite trend, which might require further study (Fig. 3). These results indicate that CAT and AsA-GSH antioxidant cycling systems play an important role in the decline of viability of *E. mollis* seeds. These results were consistent with the findings of Cheng in *Avena sativa* seeds and Wu in *Oryza sativa* seeds [67, 68].

In this study, 99 and 142 significantly DAMs were found between fresh seeds and those stored for 1 and 3 months, and 91 significantly changed DAMs were identified between seeds stored for 3 months and 1 month (Fig. 8a, b, c). Moreover, the main types of DAMs were flavonoids, amino acids and derivatives, and phenolic acids (Fig. 8d). Simultaneous qualitative and quantitative analysis of the detected key metabolites revealed that a decrease in the expression of quercetin-3-O-robinobioside, chrysoeriol-8-C-arabinoside-7-O-rutinoside, vitexin-2”-O-rhamnoside and kaempferol-3,7-O-dirhamnoside (kaempferitrin), oleamide (9-Octadecenamide), 3-Amino-1-propionic sulfonic acid, l-theanine, and creatine may lead to a decrease in seed viability of *E. mollis*, while the expression of gossypetin-3-O-rutinoside, chrysoeriol-6,8-di-C-glucoside, n-acetyl-beta-alanine, 4-pyridoxic acid-O-glucoside, 2’-hydroxy-2-methoxychalcone, 1-linoleoylglycerol* and acetryptine were increased, which may lead to the decrease in seed viability of *E. Mollis* (Fig. 9). It was indicated that flavonoids and phenolic acids play an important role in the process of seed viability decline in *E. mollis*. Flavonoids are a class of natural plant compounds with antioxidant properties that scavenge free radicals and play an effective role in detoxifying ROS [69]. Astudy of *Arabidopsis thaliana* showed that seed flavonoids were important for environmental adaptation, reactive oxygen species homeostasis, dormancy and longevity [70]. In wang’s study, a positive correlation between flavonoid content and antioxidant capacity in *Brassica napus* seeds, which was an important regulator of seed viability change [71]. It is evident that flavonoids may play a role in seed viability by participating in the regulation of oxidative metabolic homeostasis.

Tryptophan is abundant in plants, and its antioxidant effect in plant seeds has not yet been clearly reported, but in animals it has been reported that there is a link between tryptophan and the antioxidant function of the organism. In animal studies, tryptophan was found to increase the activity of antioxidant enzymes and reduce the occurrence of oxidative reactions in their bodies [72–73]. In contrast, the antioxidant function of tryptophan in plant materials has not been reported. However, the combined transcriptomic and metabolomic analyses in this study revealed that viability-related DEGs and DAMs were co-enriched in the flavonoid biosynthesis and tryptophan metabolism pathways. In the flavonoid biosynthesis pathway, six key genes were detected: *CHS*, *C4H*, *HCT*, *DFR*, *AtVSR1* and *CCoAOMT*, and the expression patterns of *CHS*, *HCT*, *CCoAOMT* and *DFR* were similar. (Fig. 12a). Among them, *CHS* is a key enzyme in the catalytic flavonoid biosynthesis pathway and plays a crucial role in maintaining seed viability [74]. Furthermore, In the tryptophan metabolism pathway five key genes were detected: *ALDH*, *NIT*, *AMIE*, *TGG* and *CAT*, moreover, *TGG*, *NIT* and *CAT* genes expression patterns were similar. (Fig. 13a). Among the aforementioned genes, the *ALDH* gene catalyzes the oxidation of reactive aldehydes to the corresponding carboxylic acids, which have a detoxifying effect on reactive oxygen species accumulated under stress conditions. Rodrigues found that the absence of *ALDH*7 in *Oryza sativa* seeds can lead to browning during storage, then resulted decreased viability [75]. In addition, the related genes *HCT*, *CCoAOMT*, *DFR*, *C4H*2, *AtVSR*1, *TGG*, *NIT*, *CAT*, and *AMIE* in the above two pathways play a significant role in the process of seed viability decline in *E. mollis*. The flavonoid biosynthesis pathway and tryptophan metabolism pathway are collectively involved in regulating the decline in viability of *E. mollis* seeds at ambient temperature. Furthermore, the antioxidant defense system appears to be the key to their function. However, the mechanism of interaction between the two pathways remains to be elucidated.

Transcription factors are important regulators of seed viability during storage. In recent years, it has been found that *WRKY*, *C3H*, *bZIP* and *B3* transcription factors play important regulatory roles in the changes of *Triticum aestivum* seed viability [24]. While in the study of *Chenopodium quinoa*, *bHLH*, *NAC*, *FAR1* and *AP2/ERF-ERF* were found to be important factors regulating the changes of seed viability [21]. But in this study, we found that *MYB-related* (10 members), *bHLH* (6 members), *AP2/ERF-ERF* (5 members), *C3H* (4 members), *NAC* (4 members), *bZIP* (2 members), *WRKY* (2 members), *MYB* (2 members) and *B3* (1 member) were important regulatory factors in the process of *E. mollis* seed viability decline (Fig. 14). These results further indicated that *WRKY*, *C3H*, *bZIP*, *B3*, *bHLH*, *NAC* and *AP2/ERF-ERF* transcription factors play an important role in regulating seed viability. In addition, previous studies have shown that *bHLH* and *MYB* participate in flavonoid biosynthesis [76]. It can be seen that *bHLH* and *MYB* transcription factors may be important factors in the regulation of *E. mollis* seed viability decline by the flavonoid biosynthesis pathway, but the specific mechanism of the role of these transcription factors needs to be investigated in depth.

## Conclusion

This study is the first to integrate physiological, transcriptomics and metabolomics to analyze the changes of *E. mollis* seed viability after different storage times in the natural state.This study initially revealed the mechanism of *E. mollis* seed viability decline. Physiological indicators show that the antioxidant defense system is involved in the regulation of seed viability changes. Transcriptomic analysis identified *WRKY*, *C3H*, *bZIP*, *B3*, *bHLH*, *NAC*, and *AP2/ERF-ERF* as key regulators during the decline of seed viability. Secondly, flavonoid biosynthesis and tryptophan metabolism pathways also affected the viability of *E. mollis* seeds, and the key antioxidant genes such as *CAT*, *ALDH*, *CHS*, and *C4H* were highly expressed. This study also showed that flavonoid biosynthesis and tryptophan metabolism participate in regulating the decline of seed viability by acting on the antioxidant defense system. In conclusion, this study clarified the potential mechanisms at the physiological and molecular level in the process of *E. mollis* seed viability decline, and these findings will provide a theoretical basis for delaying the decline of *E. mollis* seed viability and improving the conservation of *E. mollis* seed germplasm resources.

## Electronic supplementary material

Below is the link to the electronic supplementary material.

Supplementary Material 1

## Data Availability

All data generated or analyzed in the course of this study are available in Supplementary Materials and the Sequence Reading Archive (https://www.ncbi.nlm.nih.gov/sra) under accession numbers PRJNA1151851.

## Abbreviations

DAMs: Differential accumulation metabolites
DEGs: Differentially expressed genes
KEGG: Kyoto encyclopedia of genes and genomes
O_2_^−^: Superoxide anion radical
H_2_O_2_: Hydrogen peroxide
OH: Hydroxyl free radical
SOD: Superoxide dismutase
CAT: Catalase
AsA: Ascorbate acid
GSH: Glutathione
APX: Ascorbic acid peroxidase
GR: Glutathione reductase
DHAR: Dehydroascorbate reductase
MDHAR: Monodehy droascorbate reductase
