# Supplementary material for: Integrated transcriptomics and metabolomics to explore the mechanisms of Elaeagnus mollis diels seed viability decline
Source: BMC Genomics. 2025 Apr 2;26:333. doi: 10.1186/s12864-025-11483-3 (PMC11963366; doi:10.1186/s12864-025-11483-3)
Supplement: Supplementary file 1 — Supplementary Material 1 [file 12864_2025_11483_MOESM1_ESM.docx]

| **Table S1. Sample quality control** | | | | | | | |
| --- | --- | --- | --- | --- | --- | --- | --- |
| **Sample** | **Raw Reads** | **Clean Reads** | **Clean Base(G)** | **Error Rate(%)** | **Q20(%)** | **Q30(%)** | **GC Content(%)** |
| Fresh-1 | 63871416 | 61070156 | 9.16 | 0.02 | 98.34 | 94.6 | 43.49 |
| Fresh-2 | 64046886 | 61249432 | 9.19 | 0.02 | 98.38 | 94.73 | 43.44 |
| Fresh-3 | 60776620 | 57884172 | 8.68 | 0.02 | 98.23 | 94.34 | 43.44 |
| Store-1m-1 | 57972278 | 55006110 | 8.25 | 0.02 | 98.32 | 94.56 | 43.39 |
| Store-1m-2 | 54893876 | 52317804 | 7.85 | 0.03 | 98.1 | 94.09 | 43.25 |
| Store-1m-3 | 51850288 | 49458362 | 7.42 | 0.03 | 98 | 93.79 | 43.26 |
| Store-3m-1 | 53284508 | 50882654 | 7.63 | 0.02 | 98.23 | 94.47 | 43.32 |
| Store-3m-2 | 48391300 | 45921072 | 6.89 | 0.02 | 98.24 | 94.42 | 43.35 |
| Store-3m-3 | 51801844 | 49498672 | 7.42 | 0.02 | 98.22 | 94.38 | 43.39 |

| **Table S2. Conjoint analysis of DEGs and DAMs.（Store-1m_vs_Fresh_common_KEGG）** | | | | | | | | | | | | | | | |  | |  |
| --- | --- | --- | --- | --- | --- | --- | --- | --- | --- | --- | --- | --- | --- | --- | --- | --- | --- | --- |
| **KEGG_map** | **Description** | **Rich_factor_meta** | **DiffRatio_meta** | **BgRatio_meta** | **P-value_meta** | **Adjusted P-value_meta** | **Index_meta** | **CID_meta** | **Rich_factor_gene** | **DiffRatio_gene** | **BgRatio_gene** | **P-value_gene** | **Adjusted P-value_gene** | **Index_gene** | **KO_gene** | | **Hyperlink** | |
| ko01100 | Metabolic pathways | 10/207 | 10/20 | 207/328 | 0.930327015 | 1 | Lmbn002644;mws1094;Zmyn000108;pmb0064;mws0014;mws0677;mws0133;pme0490;Lmqn000780;pme3009 | C06758+C00974+C00818+C04831+C01494+C00978+C00153+C00253+C00043+C02341 | 173/9441 | 173/362 | 9441/24053 | 0.000539546 | 0.058810465 | Cluster-32694.8;Cluster-16616.3;Cluster-6384.3;Cluster-22112.3;Cluster-34140.11;Cluster-20438.4;Cluster-36336.11;Cluster-29016.0;Cluster-35358.9;Cluster-27377.0;Cluster-29771.5;Cluster-9930.2;Cluster-27606.0;Cluster-15104.0;Cluster-33239.3;Cluster-34361.4;Cluster-34305.4;Cluster-21541.4;Cluster-30519.1;Cluster-31060.12;Cluster-28939.1;Cluster-13468.3;Cluster-3618.1;Cluster-29767.1;Cluster-28349.1;Cluster-13630.2;Cluster-28023.0;Cluster-14596.2;Cluster-28392.6;Cluster-5489.2;Cluster-31888.7;Cluster-22772.4;Cluster-36259.6;Cluster-30586.3;Cluster-36968.1;Cluster-5933.0;Cluster-3393.1;Cluster-7797.0;Cluster-35792.4;Cluster-5822.0;Cluster-13468.1;Cluster-31865.0;Cluster-35249.4;Cluster-35988.4;Cluster-33604.3;Cluster-31443.5;Cluster-1127.0;Cluster-2340.1;Cluster-3050.0;Cluster-17230.2;Cluster-31060.7;Cluster-2634.0;Cluster-5540.0;Cluster-2787.0;Cluster-19408.0;Cluster-29706.6;Cluster-16484.1;Cluster-5013.0;Cluster-23603.5;Cluster-8211.0;Cluster-4011.0;Cluster-35042.20;Cluster-22138.0;Cluster-27304.0;Cluster-3393.0;Cluster-2292.4;Cluster-1428.0;Cluster-30625.2;Cluster-31214.11;Cluster-2839.0;Cluster-7194.0;Cluster-31213.18;Cluster-4785.0;Cluster-4677.4;Cluster-23265.2;Cluster-26920.2;Cluster-5535.9;Cluster-2508.0;Cluster-36303.11;Cluster-32969.0;Cluster-4642.3;Cluster-5391.0;Cluster-36828.5;Cluster-28349.2;Cluster-5709.0;Cluster-31248.1;Cluster-2698.1;Cluster-14058.3;Cluster-5744.0;Cluster-2824.0;Cluster-31077.2;Cluster-35123.4;Cluster-8319.0;Cluster-29126.2;Cluster-4711.0;Cluster-32262.2;Cluster-4376.0;Cluster-4677.6;Cluster-2714.0;Cluster-6203.0;Cluster-2969.0;Cluster-6384.2;Cluster-2483.0;Cluster-3238.0;Cluster-4441.0;Cluster-34904.11;Cluster-2924.0;Cluster-33996.0;Cluster-36548.3;Cluster-17449.1;Cluster-2849.0;Cluster-964.0;Cluster-5159.0;Cluster-20566.0;Cluster-13468.5;Cluster-14058.4;Cluster-1920.0;Cluster-2207.0;Cluster-2712.0;Cluster-2292.2;Cluster-4682.0;Cluster-20684.0;Cluster-4677.8;Cluster-5012.0;Cluster-35718.7;Cluster-36695.5;Cluster-2875.0;Cluster-6503.0;Cluster-3196.0;Cluster-31297.8;Cluster-13419.6;Cluster-26703.0;Cluster-24060.12;Cluster-906.0;Cluster-2056.0;Cluster-4110.0;Cluster-33604.13;Cluster-5271.0;Cluster-11218.3;Cluster-36229.6;Cluster-29540.0;Cluster-5489.1;Cluster-7957.7;Cluster-2333.0;Cluster-35961.1;Cluster-5489.4;Cluster-4642.4;Cluster-31897.2;Cluster-29154.0;Cluster-2798.0;Cluster-3165.0;Cluster-29462.0;Cluster-27846.2;Cluster-1548.0;Cluster-2819.1;Cluster-2184.0;Cluster-36968.5;Cluster-2292.5;Cluster-4677.5;Cluster-5804.0;Cluster-29977.5;Cluster-22226.0;Cluster-5383.0;Cluster-4397.0;Cluster-31297.9;Cluster-4006.0;Cluster-8201.0;Cluster-31241.3;Cluster-25860.5;Cluster-28939.0;Cluster-25433.0;Cluster-34140.9;Cluster-4226.0 | K03953+K00695+K01595+K01062+K11420+K01598+K01214+K09480+K01952+K12349+K13648+K05359+K12524+K01535+K01054+K01897+K01969+K01431+K01513+K03885+K00134+K00264+K00134+K18819+K05546+K01689+K13080+K00789+K23558+K01602+K00759+K00025+K13606+K00951+K01426+K02717+K05298+K01188+K10527+K00549+K00264+K06617+K01805+K01188+K01115+K00469+K03781+K01623+K14272+K18819+K03885+K00430+K02699+K08915+K07964+K02257+K03781+K08905+K19801+K01673+K01501+K00469+K12447+K15631+K05298+K01602+K00430+K05573+K02265+K10960+K08912+K11155+K00600+K08912+K00430+K01097+K15397+K03542+K16055+K07513+K01251+K00695+K01520+K05546+K00128+K08738+K00927+K01674+K02638+K02721+K11430+K01689+K03541+K01915+K00434+K00927+K03146+K08912+K01115+K01828+K10534+K01595+K02692+K02639+K01237+K00658+K02693+K00688+K03860+K15633+K08901+K00830+K01915+K13260+K00264+K01674+K14190+K02259+K08901+K01602+K00281+K10256+K08912+K09753+K01805+K00696+K02115+K01915+K02701+K02133+K22748+K01696+K01426+K02698+K00605+K01784+K01115+K04035+K01611+K04040+K01784+K01602+K00794+K00975+K01183+K01602+K01251+K02433+K20547+K02723+K01092+K01681+K03937+K02113+K01623+K14332+K01426+K01602+K08912+K02641+K05546+K10532+K01742+K00026+K02133+K01100+K00615+K00106+K01792+K00134+K01673+K11420+K00454 | | [https:\\www.genome.jp\dbget-bin\www_bget?map01100](maps\\ko01100.html) | |
| ko00380 | Tryptophan metabolism | 1/14 | 1/20 | 14/328 | 0.593160401 | 1 | mws0677 | C00978 | 9/272 | 9/362 | 272/24053 | 0.022622009 | 1 | Cluster-36968.1;Cluster-1127.0;Cluster-16484.1;Cluster-4011.0;Cluster-5709.0;Cluster-4441.0;Cluster-34904.11;Cluster-24060.12;Cluster-36968.5 | K01426+K03781+K03781+K01501+K00128+K01237+K00658+K01426+K01426 | | [https:\\www.genome.jp\dbget-bin\www_bget?map00380](maps\\ko00380.html) | |
| ko00944 | Flavone and flavonol biosynthesis | 1/15 | 1/20 | 15/328 | 0.619073751 | 1 | pme3227 | C12628 | 1/13 | 1/362 | 13/24053 | 0.178963789 | 1 | Cluster-28023.0 | K13080 | | [https:\\www.genome.jp\dbget-bin\www_bget?map00944](maps\\ko00944.html) | |
| ko01110 | Biosynthesis of secondary metabolites | 6/117 | 6/20 | 117/328 | 0.781629756 | 1 | mws1094;pmb0064;mws0014;mws0049;mws0677;pme0490 | C00974+C04831+C01494+C12127+C00978+C00253 | 80/4873 | 80/362 | 4873/24053 | 0.207216265 | 1 | Cluster-16616.3;Cluster-36336.11;Cluster-35358.9;Cluster-9930.2;Cluster-27606.0;Cluster-12849.14;Cluster-30519.1;Cluster-28939.1;Cluster-13468.3;Cluster-3618.1;Cluster-13630.2;Cluster-14596.2;Cluster-24307.6;Cluster-28553.0;Cluster-28392.6;Cluster-5489.2;Cluster-22772.4;Cluster-36259.6;Cluster-34264.6;Cluster-31518.9;Cluster-7797.0;Cluster-35792.4;Cluster-5822.0;Cluster-13468.1;Cluster-35988.4;Cluster-33604.3;Cluster-1127.0;Cluster-2340.1;Cluster-3050.0;Cluster-2634.0;Cluster-29706.6;Cluster-16484.1;Cluster-22138.0;Cluster-2292.4;Cluster-1428.0;Cluster-2839.0;Cluster-4785.0;Cluster-23265.2;Cluster-5535.9;Cluster-36303.11;Cluster-32969.0;Cluster-5391.0;Cluster-5709.0;Cluster-2698.1;Cluster-35123.4;Cluster-32262.2;Cluster-2714.0;Cluster-6203.0;Cluster-34904.11;Cluster-33996.0;Cluster-17449.1;Cluster-964.0;Cluster-20566.0;Cluster-13468.5;Cluster-1920.0;Cluster-2207.0;Cluster-2292.2;Cluster-4682.0;Cluster-5012.0;Cluster-36695.5;Cluster-26703.0;Cluster-2056.0;Cluster-33604.13;Cluster-5271.0;Cluster-36229.6;Cluster-5489.1;Cluster-7957.7;Cluster-2333.0;Cluster-5489.4;Cluster-3165.0;Cluster-29462.0;Cluster-2819.1;Cluster-2292.5;Cluster-5383.0;Cluster-4397.0;Cluster-8201.0;Cluster-31241.3;Cluster-25860.5;Cluster-28939.0;Cluster-4226.0 | K00695+K01214+K01952+K05359+K12524+K15405+K01513+K00134+K00264+K00134+K01689+K00789+K13496+K18693+K23558+K01602+K00025+K13606+K13496+K23136+K01188+K10527+K00549+K00264+K01188+K01115+K03781+K01623+K14272+K00430+K02257+K03781+K12447+K01602+K00430+K10960+K00600+K00430+K15397+K16055+K07513+K00695+K00128+K00927+K01689+K00927+K01115+K01828+K00658+K00688+K15633+K00830+K13260+K00264+K14190+K02259+K01602+K00281+K09753+K00696+K01696+K00605+K01115+K04035+K04040+K01602+K00794+K00975+K01602+K01092+K01681+K01623+K01602+K01742+K00026+K00615+K00106+K01792+K00134+K00454 | | [https:\\www.genome.jp\dbget-bin\www_bget?map01110](maps\\ko01110.html) | |
| ko00999 | Biosynthesis of various plant secondary metabolites | 1/17 | 1/20 | 17/328 | 0.666272158 | 1 | pmb0064 | C04831 | 4/195 | 4/362 | 195/24053 | 0.337966523 | 1 | Cluster-14596.2;Cluster-31518.9;Cluster-7797.0;Cluster-35988.4 | K00789+K23136+K01188+K01188 | | [https:\\www.genome.jp\dbget-bin\www_bget?map00999](maps\\ko00999.html) | |
| ko00053 | Ascorbate and aldarate metabolism | 1/9 | 1/20 | 9/328 | 0.436439831 | 1 | Zmyn000108 | C00818 | 6/339 | 6/362 | 339/24053 | 0.402167227 | 1 | Cluster-31443.5;Cluster-35042.20;Cluster-22138.0;Cluster-5709.0;Cluster-4711.0;Cluster-1920.0 | K00469+K00469+K12447+K00128+K00434+K14190 | | [https:\\www.genome.jp\dbget-bin\www_bget?map00053](maps\\ko00053.html) | |
| ko00940 | Phenylpropanoid biosynthesis | 1/11 | 1/20 | 11/328 | 0.504994596 | 1 | mws0014 | C01494 | 4/268 | 4/362 | 268/24053 | 0.57566585 | 1 | Cluster-2634.0;Cluster-1428.0;Cluster-23265.2;Cluster-5012.0 | K00430+K00430+K00430+K09753 | | [https:\\www.genome.jp\dbget-bin\www_bget?map00940](maps\\ko00940.html) | |
| ko01250 | Biosynthesis of nucleotide sugars | 1/12 | 1/20 | 12/328 | 0.536225221 | 1 | Lmqn000780 | C00043 | 7/505 | 7/362 | 505/24053 | 0.639316519 | 1 | Cluster-31443.5;Cluster-35042.20;Cluster-22138.0;Cluster-26920.2;Cluster-4110.0;Cluster-29540.0;Cluster-2333.0 | K00469+K00469+K12447+K01097+K01784+K01784+K00975 | | [https:\\www.genome.jp\dbget-bin\www_bget?map01250](maps\\ko01250.html) | |
| ko00520 | Amino sugar and nucleotide sugar metabolism | 1/11 | 1/20 | 11/328 | 0.504994596 | 1 | Lmqn000780 | C00043 | 8/639 | 8/362 | 639/24053 | 0.748816359 | 1 | Cluster-29771.5;Cluster-22138.0;Cluster-26920.2;Cluster-4110.0;Cluster-29540.0;Cluster-2333.0;Cluster-35961.1;Cluster-29154.0 | K13648+K12447+K01097+K01784+K01784+K00975+K01183+K20547 | | [https:\\www.genome.jp\dbget-bin\www_bget?map00520](maps\\ko00520.html) | |
| ko00941 | Flavonoid biosynthesis | 2/16 | 2/20 | 16/328 | 0.25396872 | 1 | mws1094;mws0049 | C00974+C12127 | 1/117 | 1/362 | 117/24053 | 0.831120775 | 1 | Cluster-28023.0 | K13080 | | [https:\\www.genome.jp\dbget-bin\www_bget?map00941](maps\\ko00941.html) | |
| ko00760 | Nicotinate and nicotinamide metabolism | 2/10 | 2/20 | 10/328 | 0.118443497 | 1 | mws0133;pme0490 | C00153+C00253 | 1/127 | 1/362 | 127/24053 | 0.854995755 | 1 | Cluster-30519.1 | K01513 | | [https:\\www.genome.jp\dbget-bin\www_bget?map00760](maps\\ko00760.html) | |
| ko00410 | beta-Alanine metabolism | 1/8 | 1/20 | 8/328 | 0.398869153 | 1 | MWS2413 | C01073 | 2/239 | 2/362 | 239/24053 | 0.877210777 | 1 | Cluster-21541.4;Cluster-5709.0 | K01431+K00128 | | [https:\\www.genome.jp\dbget-bin\www_bget?map00410](maps\\ko00410.html) | |
| ko01240 | Biosynthesis of cofactors | 2/34 | 2/20 | 34/328 | 0.637117061 | 1 | mws0133;pme0490 | C00153+C00253 | 9/994 | 9/362 | 994/24053 | 0.96559 | 1 | Cluster-20438.4;Cluster-14596.2;Cluster-29706.6;Cluster-4785.0;Cluster-5709.0;Cluster-4376.0;Cluster-1920.0;Cluster-2207.0;Cluster-7957.7 | K01598+K00789+K02257+K00600+K00128+K03146+K14190+K02259+K00794 | | [https:\\www.genome.jp\dbget-bin\www_bget?map01240](maps\\ko01240.html) | |

| **Table S3. Conjoint analysis of DEGs and DAMs. （Store-3m_vs_Fresh_common_KEGG）** | | | | | | | | | | | | | | | | |
| --- | --- | --- | --- | --- | --- | --- | --- | --- | --- | --- | --- | --- | --- | --- | --- | --- |
| **KEGG_map** | **Description** | **Rich_factor_meta** | **DiffRatio_meta** | **BgRatio_meta** | **P-value_meta** | **Adjusted P-value_meta** | **Index_meta** | **CID_meta** | **Rich_factor_gene** | **DiffRatio_gene** | **BgRatio_gene** | **P-value_gene** | **Adjusted P-value_gene** | **Index_gene** | **KO_gene** | **Hyperlink** |
| ko01100 | Metabolic pathways | 18/208 | 18/32 | 208/329 | 0.853786156 | 1 | Lmdn006025;pme1216;Lmtn004049;mws1094;ML10197929;mws1346;Lmap001823;mws0014;MWSmce118;mws0230;mws0677;pme0075;mws1179;mws1375;mws0133;mws4085;Zmfn000481;mws0675 | C12631+C10164+C06082+C00974+C00300+C00956+C05143+C01494+C02835+C00188+C00978+C00624+C09099+C05324+C00153+C00482+C00190+C00455 | 468/9441 | 468/792 | 9441/24053 | 1.70772E-30 | 2.1688E-28 | Cluster-16616.3;Cluster-5489.3;Cluster-5489.0;Cluster-28939.1;Cluster-4677.8;Cluster-5489.6;Cluster-5012.0;Cluster-4677.5;Cluster-5081.0;Cluster-5489.4;Cluster-1941.0;Cluster-2803.0;Cluster-2340.0;Cluster-5780.0;Cluster-2570.0;Cluster-8211.1;Cluster-4197.0;Cluster-389.2;Cluster-4090.0;Cluster-3285.0;Cluster-1316.0;Cluster-2474.0;Cluster-14596.2;Cluster-4677.7;Cluster-4677.2;Cluster-5091.0;Cluster-5771.0;Cluster-2726.0;Cluster-568.0;Cluster-2096.0;Cluster-2299.0;Cluster-1127.0;Cluster-7797.0;Cluster-2549.0;Cluster-1428.0;Cluster-2787.0;Cluster-16484.1;Cluster-2292.4;Cluster-2340.1;Cluster-3050.0;Cluster-23730.1;Cluster-2969.0;Cluster-5744.0;Cluster-5933.0;Cluster-5489.2;Cluster-8319.0;Cluster-29126.2;Cluster-2508.0;Cluster-33604.5;Cluster-2634.0;Cluster-4677.6;Cluster-3393.1;Cluster-4376.0;Cluster-3393.0;Cluster-4011.0;Cluster-2839.0;Cluster-5644.0;Cluster-964.0;Cluster-2698.1;Cluster-5159.0;Cluster-2483.0;Cluster-2824.0;Cluster-3238.0;Cluster-13630.2;Cluster-5540.0;Cluster-2849.0;Cluster-5822.0;Cluster-4677.4;Cluster-34140.11;Cluster-4441.0;Cluster-2924.0;Cluster-3618.1;Cluster-8211.0;Cluster-2712.0;Cluster-2207.0;Cluster-8201.0;Cluster-2292.2;Cluster-1920.0;Cluster-2184.0;Cluster-2333.0;Cluster-2875.0;Cluster-5271.0;Cluster-2798.0;Cluster-4642.4;Cluster-2056.0;Cluster-11218.3;Cluster-4110.0;Cluster-906.0;Cluster-5013.0;Cluster-2292.5;Cluster-4006.0;Cluster-7194.0;Cluster-5489.1;Cluster-3196.0;Cluster-2532.0;Cluster-2796.0;Cluster-31297.9;Cluster-3165.0;Cluster-2375.0;Cluster-4642.3;Cluster-5489.5;Cluster-17746.0;Cluster-1548.0;Cluster-17230.2;Cluster-4397.0;Cluster-6203.0;Cluster-5383.0;Cluster-2483.1;Cluster-4138.0;Cluster-2701.0;Cluster-5709.0;Cluster-2743.0;Cluster-6384.3;Cluster-3923.0;Cluster-534.0;Cluster-2361.0;Cluster-3186.0;Cluster-4393.0;Cluster-1257.0;Cluster-4441.1;Cluster-2280.0;Cluster-4226.0;Cluster-5120.0;Cluster-3895.1;Cluster-2554.0;Cluster-36373.4;Cluster-28617.10;Cluster-3325.0;Cluster-1968.0;Cluster-3798.0;Cluster-5450.0;Cluster-2284.0;Cluster-8263.0;Cluster-1447.0;Cluster-4194.0;Cluster-4338.0;Cluster-3895.0;Cluster-2690.0;Cluster-3620.0;Cluster-17333.0;Cluster-2257.0;Cluster-4682.0;Cluster-5771.1;Cluster-2479.0;Cluster-22772.4;Cluster-7525.0;Cluster-629.0;Cluster-1676.0;Cluster-428.0;Cluster-5250.0;Cluster-4359.0;Cluster-2553.0;Cluster-6503.0;Cluster-4785.0;Cluster-2079.0;Cluster-468.0;Cluster-2822.0;Cluster-3522.0;Cluster-552.0;Cluster-2451.0;Cluster-291.0;Cluster-6778.0;Cluster-2428.0;Cluster-2110.0;Cluster-1594.0;Cluster-2613.0;Cluster-2727.0;Cluster-26651.7;Cluster-1692.0;Cluster-1005.0;Cluster-4677.0;Cluster-2819.0;Cluster-19779.2;Cluster-2378.0;Cluster-1315.0;Cluster-7790.0;Cluster-2721.0;Cluster-2723.0;Cluster-4223.0;Cluster-1962.0;Cluster-24413.0;Cluster-3599.0;Cluster-5765.0;Cluster-4955.0;Cluster-25728.0;Cluster-6869.0;Cluster-5867.0;Cluster-3272.0;Cluster-29016.0;Cluster-2246.0;Cluster-771.0;Cluster-3890.1;Cluster-3735.0;Cluster-19634.0;Cluster-28939.0;Cluster-5189.0;Cluster-2309.0;Cluster-5391.0;Cluster-32502.5;Cluster-3427.0;Cluster-4066.0;Cluster-4711.0;Cluster-1953.0;Cluster-1460.0;Cluster-817.0;Cluster-398.0;Cluster-5974.0;Cluster-1746.0;Cluster-19634.1;Cluster-1210.0;Cluster-2714.0;Cluster-597.0;Cluster-3090.0;Cluster-224.0;Cluster-21201.1;Cluster-2360.0;Cluster-4677.1;Cluster-392.0;Cluster-2710.0;Cluster-7407.0;Cluster-840.0;Cluster-23136.2;Cluster-1235.0;Cluster-570.0;Cluster-4247.0;Cluster-4507.0;Cluster-2477.0;Cluster-3335.0;Cluster-6203.2;Cluster-5442.0;Cluster-2044.0;Cluster-3403.0;Cluster-29989.0;Cluster-33543.8;Cluster-32502.0;Cluster-859.0;Cluster-3331.0;Cluster-3363.0;Cluster-3127.0;Cluster-1786.0;Cluster-2224.0;Cluster-3280.0;Cluster-27606.0;Cluster-21201.0;Cluster-2887.0;Cluster-2053.0;Cluster-36259.6;Cluster-12996.7;Cluster-6203.1;Cluster-4323.0;Cluster-4243.0;Cluster-1495.0;Cluster-5253.0;Cluster-2374.0;Cluster-29934.2;Cluster-3716.0;Cluster-2842.0;Cluster-3037.0;Cluster-5535.3;Cluster-1162.0;Cluster-2520.0;Cluster-1139.0;Cluster-3034.0;Cluster-27377.0;Cluster-3060.0;Cluster-2819.1;Cluster-2675.0;Cluster-2009.1;Cluster-3451.0;Cluster-22462.0;Cluster-4986.0;Cluster-2100.0;Cluster-1898.0;Cluster-12996.6;Cluster-4667.0;Cluster-5540.2;Cluster-4383.0;Cluster-841.0;Cluster-1877.0;Cluster-2851.0;Cluster-4269.0;Cluster-5804.0;Cluster-2141.0;Cluster-30625.2;Cluster-13468.1;Cluster-2543.0;Cluster-1265.0;Cluster-2758.0;Cluster-3600.0;Cluster-3689.0;Cluster-2810.0;Cluster-17449.1;Cluster-3100.0;Cluster-11667.1;Cluster-1559.0;Cluster-3273.0;Cluster-2928.0;Cluster-5383.2;Cluster-2010.0;Cluster-25647.0;Cluster-3618.0;Cluster-3194.0;Cluster-10444.0;Cluster-539.0;Cluster-910.0;Cluster-2404.0;Cluster-2649.0;Cluster-2711.0;Cluster-1321.0;Cluster-24060.11;Cluster-2107.0;Cluster-4595.0;Cluster-268.0;Cluster-2656.0;Cluster-30960.0;Cluster-19779.0;Cluster-21523.1;Cluster-3215.0;Cluster-2420.0;Cluster-4662.0;Cluster-2896.0;Cluster-4032.0;Cluster-2367.0;Cluster-35698.0;Cluster-1926.0;Cluster-2889.0;Cluster-34845.12;Cluster-4406.0;Cluster-2313.0;Cluster-2310.0;Cluster-15662.0;Cluster-27616.1;Cluster-955.0;Cluster-4782.0;Cluster-23136.1;Cluster-2510.0;Cluster-4867.0;Cluster-4890.0;Cluster-5018.0;Cluster-2443.0;Cluster-744.0;Cluster-2172.0;Cluster-21523.2;Cluster-2663.0;Cluster-11158.1;Cluster-2686.0;Cluster-3723.0;Cluster-13629.0;Cluster-35212.1;Cluster-4072.0;Cluster-32581.2;Cluster-2871.0;Cluster-29362.0;Cluster-2985.0;Cluster-2645.0;Cluster-5658.0;Cluster-3237.0;Cluster-23050.0;Cluster-28392.6;Cluster-29462.0;Cluster-822.0;Cluster-15104.0;Cluster-3655.0;Cluster-9930.2;Cluster-21541.4;Cluster-2442.0;Cluster-956.0;Cluster-10586.0;Cluster-22375.2;Cluster-34071.0;Cluster-2523.0;Cluster-1961.0;Cluster-5083.0;Cluster-2415.0;Cluster-3688.0;Cluster-3143.0;Cluster-7227.0;Cluster-20566.0;Cluster-36336.16;Cluster-5044.0;Cluster-28983.0;Cluster-1885.0;Cluster-14058.4;Cluster-13732.1;Cluster-14670.0;Cluster-33164.3;Cluster-32103.0;Cluster-8110.0;Cluster-10641.0;Cluster-31888.6;Cluster-34305.4;Cluster-29924.0;Cluster-22112.3;Cluster-8359.0;Cluster-6021.0;Cluster-31241.3;Cluster-31214.11;Cluster-33410.0;Cluster-31443.5;Cluster-6965.0;Cluster-31865.0;Cluster-36303.11;Cluster-22263.3;Cluster-19408.0;Cluster-14097.0;Cluster-13468.5;Cluster-31402.1;Cluster-5134.0;Cluster-26651.4;Cluster-32695.0;Cluster-31060.7;Cluster-32730.4;Cluster-29977.5;Cluster-9655.0;Cluster-5803.0;Cluster-22138.0;Cluster-29868.0;Cluster-30445.3;Cluster-13468.3;Cluster-33368.0;Cluster-31297.8;Cluster-13419.6;Cluster-21082.1;Cluster-26068.7;Cluster-24366.0;Cluster-21523.4;Cluster-5535.10;Cluster-6384.2;Cluster-5215.5;Cluster-34657.3;Cluster-24349.1;Cluster-28349.2;Cluster-33239.3;Cluster-20684.0;Cluster-26002.4;Cluster-34074.9;Cluster-23496.0;Cluster-34153.2;Cluster-26817.1;Cluster-29540.0;Cluster-35358.9;Cluster-14058.1;Cluster-32297.0;Cluster-23150.1;Cluster-31060.12;Cluster-14058.3;Cluster-36622.0;Cluster-27846.2;Cluster-36526.0;Cluster-36741.11;Cluster-13451.0;Cluster-23224.1;Cluster-18281.0;Cluster-35358.10;Cluster-35249.4;Cluster-32470.6;Cluster-30270.0;Cluster-4454.2;Cluster-22472.2;Cluster-26868.4;Cluster-24103.0;Cluster-33604.13;Cluster-34595.10;Cluster-34062.6;Cluster-31733.5;Cluster-5535.4;Cluster-26724.0;Cluster-6098.0 | K00695+K01602+K01602+K00134+K08912+K01602+K09753+K08912+K03781+K01602+K08916+K08910+K01623+K08909+K08914+K01673+K00688+K00281+K08907+K08915+K08917+K11517+K00789+K08912+K08912+K02716+K08913+K08908+K05298+K03403+K02694+K03781+K01188+K00600+K00430+K08915+K03781+K01602+K01623+K14272+K00615+K10534+K02638+K02717+K01602+K03541+K01915+K03542+K01115+K00430+K08912+K05298+K03146+K05298+K01501+K10960+K00855+K00830+K00927+K01915+K02692+K02721+K02639+K01689+K02699+K08901+K00549+K08912+K11420+K01237+K02693+K00134+K01673+K08901+K02259+K00615+K01602+K14190+K14332+K00975+K02115+K04035+K02723+K01251+K00605+K01611+K01784+K02698+K08905+K01602+K01100+K08912+K01602+K02701+K02641+K01637+K02133+K01092+K00025+K01251+K01602+K00031+K02113+K18819+K00026+K01828+K01742+K02692+K02109+K01757+K00128+K01828+K01595+K03146+K07151+K00799+K02695+K00434+K01092+K01237+K18857+K00454+K00430+K01535+K03841+K01426+K00128+K13811+K13064+K00799+K02636+K03527+K07151+K00889+K08726+K01623+K01535+K01051+K02145+K10525+K01179+K00281+K08913+K01803+K00025+K01689+K09840+K01595+K02492+K00789+K03860+K01783+K01915+K00600+K02695+K00392+K19269+K00588+K01828+K13679+K09838+K07151+K01611+K08910+K01051+K01580+K01803+K01190+K02639+K01177+K08912+K01623+K10256+K05933+K00366+K13034+K08909+K02437+K01662+K13513+K00430+K22133+K00434+K01580+K01938+K00799+K00218+K00021+K09480+K00053+K01739+K00789+K15397+K00799+K00134+K11517+K05907+K00695+K00789+K00434+K13382+K00434+K03526+K01652+K03844+K00799+K00029+K02641+K00799+K01738+K01115+K00927+K00382+K03405+K00799+K03146+K08912+K02437+K22133+K00679+K00789+K00432+K18819+K12502+K02716+K01568+K01803+K00615+K01828+K00122+K00830+K08232+K20547+K11808+K00789+K01179+K10256+K00128+K00430+K01703+K00026+K01115+K12524+K00799+K05298+K02291+K13606+K10256+K01828+K07151+K00297+K13082+K01759+K09828+K00074+K01673+K00432+K01919+K15397+K01648+K00855+K01858+K00696+K12349+K01623+K01623+K00029+K03541+K15398+K00487+K05933+K00051+K02136+K10256+K03428+K02699+K00264+K00083+K00026+K02144+K15730+K02641+K08916+K05573+K00264+K01738+K22395+K01845+K00940+K00660+K01177+K15633+K15397+K22374+K00660+K15893+K00695+K01742+K12309+K09837+K00134+K01681+K01051+K00029+K05933+K00434+K21926+K00487+K01115+K01426+K08232+K07151+K02147+K02639+K22395+K10256+K00549+K12450+K09840+K13082+K13066+K10525+K13600+K00873+K02638+K21888+K01110+K02150+K08907+K13065+K00679+K00036+K02692+K02636+K00432+K22849+K05298+K02716+K00815+K00547+K03955+K00121+K00549+K08908+K05894+K13229+K01611+K13379+K00940+K00109+K19892+K02155+K00083+K01759+K00109+K12448+K11818+K01231+K23558+K01681+K06215+K01535+K00975+K05359+K01431+K06215+K08679+K02717+K00627+K05933+K01807+K00927+K13679+K01738+K08915+K00236+K00053+K13260+K01214+K00927+K03527+K00975+K01674+K00789+K22133+K01653+K00020+K00799+K01255+K00759+K01969+K00122+K01062+K01051+K00434+K00106+K02265+K08678+K00469+K02134+K06617+K16055+K01638+K07964+K00036+K00264+K01968+K01915+K01190+K01950+K03885+K03644+K05546+K00856+K10534+K12447+K03955+K03841+K00264+K01115+K02133+K22748+K05894+K00655+K00058+K00549+K15397+K01595+K11420+K01859+K01092+K05546+K01054+K10256+K11420+K08726+K00921+K01765+K09838+K01784+K01952+K01674+K00430+K00128+K03885+K01674+K01610+K03937+K08081+K06133+K02145+K16055+K20547+K01952+K01805+K03146+K01904+K01681+K13510+K08232+K00264+K01115+K03879+K01662+K01426+K15397+K03921+K00131 | [https:\\www.genome.jp\dbget-bin\www_bget?map01100](maps\\ko01100.html) |
| ko01110 | Biosynthesis of secondary metabolites | 12/117 | 12/32 | 117/329 | 0.475352901 | 1 | Lmdn006025;pmb3074;Lmtn004049;mws1094;mws1346;Lmap001823;mws0014;mws0230;mws0677;pme0075;mws1375;mws4085 | C12631+C12208+C06082+C00974+C00956+C05143+C01494+C00188+C00978+C00624+C05324+C00482 | 245/4873 | 245/792 | 4873/24053 | 3.4259E-13 | 4.35089E-11 | Cluster-16616.3;Cluster-5489.3;Cluster-5489.0;Cluster-28939.1;Cluster-5489.6;Cluster-5012.0;Cluster-5081.0;Cluster-5489.4;Cluster-2340.0;Cluster-4197.0;Cluster-389.2;Cluster-2474.0;Cluster-14596.2;Cluster-2096.0;Cluster-1127.0;Cluster-7797.0;Cluster-2549.0;Cluster-1428.0;Cluster-16484.1;Cluster-2292.4;Cluster-2340.1;Cluster-3050.0;Cluster-23730.1;Cluster-5489.2;Cluster-33604.5;Cluster-2634.0;Cluster-2839.0;Cluster-964.0;Cluster-2698.1;Cluster-13630.2;Cluster-5822.0;Cluster-3618.1;Cluster-2207.0;Cluster-8201.0;Cluster-2292.2;Cluster-1920.0;Cluster-2333.0;Cluster-5271.0;Cluster-2056.0;Cluster-2292.5;Cluster-5489.1;Cluster-2796.0;Cluster-3165.0;Cluster-2375.0;Cluster-5489.5;Cluster-17746.0;Cluster-4397.0;Cluster-6203.0;Cluster-5383.0;Cluster-2701.0;Cluster-5709.0;Cluster-2743.0;Cluster-1257.0;Cluster-2280.0;Cluster-4226.0;Cluster-26267.0;Cluster-5120.0;Cluster-2554.0;Cluster-28617.10;Cluster-3325.0;Cluster-1968.0;Cluster-2284.0;Cluster-4338.0;Cluster-28553.0;Cluster-17333.0;Cluster-12379.0;Cluster-4682.0;Cluster-2479.0;Cluster-22772.4;Cluster-7525.0;Cluster-629.0;Cluster-4562.0;Cluster-428.0;Cluster-5250.0;Cluster-2553.0;Cluster-4785.0;Cluster-2822.0;Cluster-3522.0;Cluster-552.0;Cluster-2451.0;Cluster-291.0;Cluster-2613.0;Cluster-2727.0;Cluster-1005.0;Cluster-2819.0;Cluster-2378.0;Cluster-7790.0;Cluster-2723.0;Cluster-4223.0;Cluster-1962.0;Cluster-24413.0;Cluster-4955.0;Cluster-5867.0;Cluster-3272.0;Cluster-2246.0;Cluster-771.0;Cluster-3890.1;Cluster-3735.0;Cluster-28939.0;Cluster-5189.0;Cluster-5391.0;Cluster-32502.5;Cluster-4066.0;Cluster-1953.0;Cluster-1460.0;Cluster-19592.0;Cluster-1210.0;Cluster-2714.0;Cluster-597.0;Cluster-3090.0;Cluster-224.0;Cluster-392.0;Cluster-840.0;Cluster-570.0;Cluster-4507.0;Cluster-2477.0;Cluster-3335.0;Cluster-1471.0;Cluster-6203.2;Cluster-2044.0;Cluster-33543.8;Cluster-32502.0;Cluster-3363.0;Cluster-3127.0;Cluster-1786.0;Cluster-2224.0;Cluster-3280.0;Cluster-27606.0;Cluster-2053.0;Cluster-36259.6;Cluster-6203.1;Cluster-1495.0;Cluster-2374.0;Cluster-5535.3;Cluster-1162.0;Cluster-1139.0;Cluster-3034.0;Cluster-31688.0;Cluster-408.0;Cluster-3060.0;Cluster-2819.1;Cluster-22462.0;Cluster-4986.0;Cluster-4667.0;Cluster-4383.0;Cluster-841.0;Cluster-1877.0;Cluster-13468.1;Cluster-2543.0;Cluster-1265.0;Cluster-2758.0;Cluster-3600.0;Cluster-3689.0;Cluster-2810.0;Cluster-17449.1;Cluster-3100.0;Cluster-11667.1;Cluster-21370.0;Cluster-1559.0;Cluster-3273.0;Cluster-2928.0;Cluster-5383.2;Cluster-25647.0;Cluster-12849.14;Cluster-3618.0;Cluster-3194.0;Cluster-910.0;Cluster-2649.0;Cluster-2711.0;Cluster-1321.0;Cluster-30960.0;Cluster-21523.1;Cluster-2420.0;Cluster-4662.0;Cluster-2896.0;Cluster-4032.0;Cluster-2367.0;Cluster-35698.0;Cluster-3226.0;Cluster-2310.0;Cluster-27616.1;Cluster-5018.0;Cluster-2443.0;Cluster-2172.0;Cluster-21523.2;Cluster-11158.1;Cluster-2686.0;Cluster-35212.1;Cluster-29362.0;Cluster-22520.12;Cluster-34264.6;Cluster-3237.0;Cluster-28392.6;Cluster-20673.0;Cluster-29462.0;Cluster-3655.0;Cluster-9930.2;Cluster-22375.2;Cluster-34071.0;Cluster-2523.0;Cluster-1961.0;Cluster-5083.0;Cluster-2415.0;Cluster-3143.0;Cluster-7227.0;Cluster-20566.0;Cluster-36336.16;Cluster-5044.0;Cluster-28983.0;Cluster-1885.0;Cluster-13732.1;Cluster-33164.3;Cluster-31241.3;Cluster-36303.11;Cluster-22263.3;Cluster-14097.0;Cluster-13468.5;Cluster-26135.3;Cluster-31197.4;Cluster-22138.0;Cluster-30445.3;Cluster-13468.3;Cluster-33368.0;Cluster-21082.1;Cluster-26068.7;Cluster-24366.0;Cluster-21523.4;Cluster-5535.10;Cluster-34657.3;Cluster-24349.1;Cluster-26817.1;Cluster-35358.9;Cluster-32297.0;Cluster-23150.1;Cluster-36622.0;Cluster-36526.0;Cluster-23224.1;Cluster-35358.10;Cluster-30270.0;Cluster-4454.2;Cluster-24103.0;Cluster-33604.13;Cluster-34062.6;Cluster-5535.4;Cluster-24307.6 | K00695+K01602+K01602+K00134+K01602+K09753+K03781+K01602+K01623+K00688+K00281+K11517+K00789+K03403+K03781+K01188+K00600+K00430+K03781+K01602+K01623+K14272+K00615+K01602+K01115+K00430+K10960+K00830+K00927+K01689+K00549+K00134+K02259+K00615+K01602+K14190+K00975+K04035+K00605+K01602+K01602+K01637+K01092+K00025+K01602+K00031+K00026+K01828+K01742+K01757+K00128+K01828+K01092+K18857+K00454+K16040+K00430+K03841+K00128+K13811+K13064+K03527+K01623+K18693+K10525+K15095+K00281+K01803+K00025+K01689+K09840+K15404+K02492+K00789+K01783+K00600+K19269+K00588+K01828+K13679+K09838+K01580+K01803+K01177+K01623+K05933+K13034+K02437+K01662+K13513+K00430+K01580+K00218+K00021+K00053+K01739+K00789+K15397+K00134+K11517+K00695+K00789+K13382+K03526+K01652+K23452+K01738+K01115+K00927+K00382+K03405+K02437+K00789+K12502+K01568+K01803+K00615+K12156+K01828+K00830+K11808+K00789+K00128+K00430+K01703+K00026+K01115+K12524+K02291+K13606+K01828+K13082+K09828+K15397+K01648+K01858+K00696+K15472+K15741+K01623+K01623+K00487+K05933+K03428+K00264+K00083+K00026+K00264+K01738+K22395+K01845+K00940+K00660+K01177+K15633+K15397+K22374+K15472+K00660+K15893+K00695+K01742+K09837+K15405+K00134+K01681+K05933+K21926+K00487+K01115+K22395+K00549+K09840+K13082+K13066+K10525+K13600+K00873+K12154+K13065+K00036+K00815+K00547+K00121+K00549+K05894+K13229+K00940+K00083+K12742+K13496+K11818+K23558+K15472+K01681+K00975+K05359+K00627+K05933+K01807+K00927+K13679+K01738+K00236+K00053+K13260+K01214+K00927+K03527+K00975+K00789+K01653+K00106+K16055+K01638+K00036+K00264+K00006+K11778+K12447+K03841+K00264+K01115+K05894+K00655+K00058+K00549+K15397+K01859+K01092+K09838+K01952+K00430+K00128+K01610+K08081+K16055+K01952+K01904+K01681+K00264+K01115+K01662+K15397+K13496 | [https:\\www.genome.jp\dbget-bin\www_bget?map01110](maps\\ko01110.html) |
| ko01230 | Biosynthesis of amino acids | 3/39 | 3/32 | 39/329 | 0.762575653 | 1 | mws1346;mws0230;pme0075 | C00956+C00188+C00624 | 66/1031 | 66/792 | 1031/24053 | 2.0999E-07 | 2.66687E-05 | Cluster-28939.1;Cluster-2340.0;Cluster-14596.2;Cluster-2549.0;Cluster-2340.1;Cluster-3050.0;Cluster-23730.1;Cluster-29126.2;Cluster-2698.1;Cluster-5159.0;Cluster-13630.2;Cluster-5822.0;Cluster-3618.1;Cluster-8201.0;Cluster-17746.0;Cluster-4338.0;Cluster-2479.0;Cluster-7525.0;Cluster-5250.0;Cluster-2553.0;Cluster-6503.0;Cluster-4785.0;Cluster-2727.0;Cluster-2819.0;Cluster-7790.0;Cluster-2246.0;Cluster-771.0;Cluster-3890.1;Cluster-28939.0;Cluster-32502.5;Cluster-1460.0;Cluster-1210.0;Cluster-597.0;Cluster-840.0;Cluster-2477.0;Cluster-3335.0;Cluster-32502.0;Cluster-1786.0;Cluster-27606.0;Cluster-3060.0;Cluster-2819.1;Cluster-4383.0;Cluster-13468.1;Cluster-2543.0;Cluster-17449.1;Cluster-3618.0;Cluster-3194.0;Cluster-21523.1;Cluster-35698.0;Cluster-21523.2;Cluster-29462.0;Cluster-9930.2;Cluster-2523.0;Cluster-1961.0;Cluster-2415.0;Cluster-7227.0;Cluster-5044.0;Cluster-13732.1;Cluster-33164.3;Cluster-13468.5;Cluster-5134.0;Cluster-13468.3;Cluster-24366.0;Cluster-21523.4;Cluster-4454.2;Cluster-24103.0 | K00134+K01623+K00789+K00600+K01623+K14272+K00615+K01915+K00927+K01915+K01689+K00549+K00134+K00615+K00031+K01623+K01803+K01689+K00789+K01783+K01915+K00600+K01803+K01623+K13034+K00053+K01739+K00789+K00134+K00789+K01652+K01738+K00927+K00789+K01803+K00615+K00789+K01703+K12524+K01623+K01623+K00264+K00264+K01738+K15633+K00134+K01681+K00549+K00873+K00549+K01681+K05359+K01807+K00927+K01738+K00053+K00927+K00789+K01653+K00264+K01915+K00264+K00058+K00549+K01681+K00264 | [https:\\www.genome.jp\dbget-bin\www_bget?map01230](maps\\ko01230.html) |
| ko00270 | Cysteine and methionine metabolism | 1/13 | 1/32 | 13/329 | 0.742434227 | 1 | mws1375 | C05324 | 35/495 | 35/792 | 495/24053 | 2.13255E-05 | 0.002708332 | Cluster-14596.2;Cluster-5822.0;Cluster-4642.4;Cluster-11218.3;Cluster-2375.0;Cluster-4642.3;Cluster-4397.0;Cluster-22772.4;Cluster-5250.0;Cluster-2428.0;Cluster-2378.0;Cluster-7790.0;Cluster-771.0;Cluster-3890.1;Cluster-32502.5;Cluster-1210.0;Cluster-840.0;Cluster-32502.0;Cluster-2224.0;Cluster-27606.0;Cluster-3037.0;Cluster-4986.0;Cluster-1877.0;Cluster-2543.0;Cluster-910.0;Cluster-21523.1;Cluster-5018.0;Cluster-2443.0;Cluster-21523.2;Cluster-3723.0;Cluster-34071.0;Cluster-2415.0;Cluster-13732.1;Cluster-24366.0;Cluster-21523.4 | K00789+K00549+K01251+K01611+K00025+K01251+K00026+K00025+K00789+K01611+K05933+K13034+K01739+K00789+K00789+K01738+K00789+K00789+K00026+K12524+K01919+K05933+K00026+K01738+K05933+K00549+K00815+K00547+K00549+K01611+K05933+K01738+K00789+K00058+K00549 | [https:\\www.genome.jp\dbget-bin\www_bget?map00270](maps\\ko00270.html) |
| ko00940 | Phenylpropanoid biosynthesis | 4/11 | 4/32 | 11/329 | 0.015034034 | 0.601361373 | pmb3074;mws0014;mws4085;mws0011 | C12208+C01494+C00482+C01533 | 18/268 | 18/792 | 268/24053 | 0.00354336 | 0.450006716 | Cluster-5012.0;Cluster-1428.0;Cluster-2634.0;Cluster-5120.0;Cluster-1968.0;Cluster-3522.0;Cluster-24413.0;Cluster-3127.0;Cluster-22462.0;Cluster-841.0;Cluster-1265.0;Cluster-2711.0;Cluster-30960.0;Cluster-2896.0;Cluster-2310.0;Cluster-29362.0;Cluster-32297.0;Cluster-30270.0 | K09753+K00430+K00430+K00430+K13064+K00588+K00430+K00430+K00487+K00083+K22395+K00487+K22395+K13066+K13065+K00083+K00430+K01904 | [https:\\www.genome.jp\dbget-bin\www_bget?map00940](maps\\ko00940.html) |
| ko00260 | Glycine, serine and threonine metabolism | 2/12 | 2/32 | 12/329 | 0.328862784 | 1 | ML10197929;mws0230 | C00300+C00188 | 15/238 | 15/792 | 238/24053 | 0.012741199 | 1 | Cluster-389.2;Cluster-2549.0;Cluster-3050.0;Cluster-964.0;Cluster-2056.0;Cluster-4682.0;Cluster-4785.0;Cluster-2723.0;Cluster-3090.0;Cluster-392.0;Cluster-2044.0;Cluster-27606.0;Cluster-17449.1;Cluster-3273.0;Cluster-24366.0 | K00281+K00600+K14272+K00830+K00605+K00281+K00600+K02437+K00382+K02437+K00830+K12524+K15633+K15893+K00058 | [https:\\www.genome.jp\dbget-bin\www_bget?map00260](maps\\ko00260.html) |
| ko00941 | Flavonoid biosynthesis | 3/16 | 3/32 | 16/329 | 0.195526384 | 1 | pmb3074;mws1094;mws1179 | C12208+C00974+C09099 | 9/117 | 9/792 | 117/24053 | 0.015316263 | 1 | Cluster-3522.0;Cluster-1495.0;Cluster-22462.0;Cluster-3689.0;Cluster-1559.0;Cluster-2711.0;Cluster-4662.0;Cluster-2310.0;Cluster-34657.3 | K00588+K13082+K00487+K00660+K00660+K00487+K13082+K13065+K01859 | [https:\\www.genome.jp\dbget-bin\www_bget?map00941](maps\\ko00941.html) |
| ko00380 | Tryptophan metabolism | 2/14 | 2/32 | 14/329 | 0.403360299 | 1 | pme1216;mws0677 | C10164+C00978 | 16/272 | 16/792 | 272/24053 | 0.018725289 | 1 | Cluster-5081.0;Cluster-1127.0;Cluster-16484.1;Cluster-4011.0;Cluster-4441.0;Cluster-5709.0;Cluster-4441.1;Cluster-36373.4;Cluster-28617.10;Cluster-3090.0;Cluster-3363.0;Cluster-24060.11;Cluster-2896.0;Cluster-3237.0;Cluster-23150.1;Cluster-31733.5 | K03781+K03781+K03781+K01501+K01237+K00128+K01237+K01426+K00128+K00382+K00128+K01426+K13066+K11818+K00128+K01426 | [https:\\www.genome.jp\dbget-bin\www_bget?map00380](maps\\ko00380.html) |
| ko01210 | 2-Oxocarboxylic acid metabolism | 2/21 | 2/32 | 21/329 | 0.628195349 | 1 | mws1346;pme0075 | C00956+C00624 | 14/255 | 14/792 | 255/24053 | 0.043692659 | 1 | Cluster-3050.0;Cluster-17746.0;Cluster-2246.0;Cluster-1460.0;Cluster-1471.0;Cluster-1786.0;Cluster-408.0;Cluster-3194.0;Cluster-3226.0;Cluster-3237.0;Cluster-29462.0;Cluster-7227.0;Cluster-33164.3;Cluster-4454.2 | K14272+K00031+K00053+K01652+K12156+K01703+K15741+K01681+K12154+K11818+K01681+K00053+K01653+K01681 | [https:\\www.genome.jp\dbget-bin\www_bget?map01210](maps\\ko01210.html) |
| ko00945 | Stilbenoid, diarylheptanoid and gingerol biosynthesis | 1/3 | 1/32 | 3/329 | 0.265058651 | 1 | pmb3074 | C12208 | 5/60 | 5/792 | 60/24053 | 0.047283147 | 1 | Cluster-26267.0;Cluster-3522.0;Cluster-22462.0;Cluster-2711.0;Cluster-2310.0 | K16040+K00588+K00487+K00487+K13065 | [https:\\www.genome.jp\dbget-bin\www_bget?map00945](maps\\ko00945.html) |
| ko00565 | Ether lipid metabolism | 1/1 | 1/32 | 1/329 | 0.097264438 | 1 | mws0120 | C00670 | 8/128 | 8/792 | 128/24053 | 0.06122614 | 1 | Cluster-33604.5;Cluster-2714.0;Cluster-3280.0;Cluster-1321.0;Cluster-22112.3;Cluster-33368.0;Cluster-22472.2;Cluster-33604.13 | K01115+K01115+K01115+K01115+K01062+K01115+K13510+K01115 | [https:\\www.genome.jp\dbget-bin\www_bget?map00565](maps\\ko00565.html) |
| ko00999 | Biosynthesis of various plant secondary metabolites | 1/17 | 1/32 | 17/329 | 0.83232175 | 1 | mws1375 | C05324 | 10/195 | 10/792 | 195/24053 | 0.11144243 | 1 | Cluster-14596.2;Cluster-7797.0;Cluster-5250.0;Cluster-3890.1;Cluster-32502.5;Cluster-840.0;Cluster-32502.0;Cluster-11667.1;Cluster-2686.0;Cluster-13732.1 | K00789+K01188+K00789+K00789+K00789+K00789+K00789+K22374+K13229+K00789 | [https:\\www.genome.jp\dbget-bin\www_bget?map00999](maps\\ko00999.html) |
| ko00290 | Valine, leucine and isoleucine biosynthesis | 1/9 | 1/32 | 9/329 | 0.606604021 | 1 | mws0230 | C00188 | 5/79 | 5/792 | 79/24053 | 0.119076651 | 1 | Cluster-2246.0;Cluster-1460.0;Cluster-1786.0;Cluster-7227.0;Cluster-33164.3 | K00053+K01652+K01703+K00053+K01653 | [https:\\www.genome.jp\dbget-bin\www_bget?map00290](maps\\ko00290.html) |
| ko00860 | Porphyrin metabolism | 1/4 | 1/32 | 4/329 | 0.337200133 | 1 | mws0230 | C00188 | 11/229 | 11/792 | 229/24053 | 0.136611412 | 1 | Cluster-2096.0;Cluster-2839.0;Cluster-2207.0;Cluster-5271.0;Cluster-428.0;Cluster-5867.0;Cluster-224.0;Cluster-36259.6;Cluster-4667.0;Cluster-2758.0;Cluster-2367.0 | K03403+K10960+K02259+K04035+K02492+K00218+K03405+K13606+K03428+K01845+K13600 | [https:\\www.genome.jp\dbget-bin\www_bget?map00860](maps\\ko00860.html) |
| ko00906 | Carotenoid biosynthesis | 1/1 | 1/32 | 1/329 | 0.097264438 | 1 | Lmtn004049 | C06082 | 6/106 | 6/792 | 106/24053 | 0.13730047 | 1 | Cluster-629.0;Cluster-291.0;Cluster-2053.0;Cluster-25647.0;Cluster-2420.0;Cluster-26817.1 | K09840+K09838+K02291+K09837+K09840+K09838 | [https:\\www.genome.jp\dbget-bin\www_bget?map00906](maps\\ko00906.html) |
| ko00460 | Cyanoamino acid metabolism | 1/8 | 1/32 | 8/329 | 0.563044605 | 1 | Lmap001823 | C05143 | 5/113 | 5/792 | 113/24053 | 0.315720666 | 1 | Cluster-7797.0;Cluster-2549.0;Cluster-4011.0;Cluster-4785.0;Cluster-7790.0 | K01188+K00600+K01501+K00600+K13034 | [https:\\www.genome.jp\dbget-bin\www_bget?map00460](maps\\ko00460.html) |
| ko02010 | ABC transporters | 2/39 | 2/32 | 39/329 | 0.917447628 | 1 | mws0230;Zmdp000972 | C00188+C22040 | 15/408 | 15/792 | 408/24053 | 0.3682809 | 1 | Cluster-24190.6;Cluster-24350.5;Cluster-3708.0;Cluster-5362.10;Cluster-5005.0;Cluster-28489.1;Cluster-30342.2;Cluster-16542.0;Cluster-24350.0;Cluster-35887.2;Cluster-5417.69;Cluster-34218.0;Cluster-10836.8;Cluster-22333.19;Cluster-22763.3 | K05665+K08711+K08711+K08711+K08711+K05666+K05666+K08711+K08711+K05666+K08711+K08711+K08711+K08711+K05666 | [https:\\www.genome.jp\dbget-bin\www_bget?map02010](maps\\ko02010.html) |
| ko00261 | Monobactam biosynthesis | 1/4 | 1/32 | 4/329 | 0.337200133 | 1 | mws0230 | C00188 | 2/43 | 2/792 | 43/24053 | 0.416140468 | 1 | Cluster-3325.0;Cluster-27606.0 | K13811+K12524 | [https:\\www.genome.jp\dbget-bin\www_bget?map00261](maps\\ko00261.html) |
| ko00220 | Arginine biosynthesis | 1/9 | 1/32 | 9/329 | 0.606604021 | 1 | pme0075 | C00624 | 5/130 | 5/792 | 130/24053 | 0.426830799 | 1 | Cluster-3050.0;Cluster-29126.2;Cluster-5159.0;Cluster-6503.0;Cluster-5134.0 | K14272+K01915+K01915+K01915+K01915 | [https:\\www.genome.jp\dbget-bin\www_bget?map00220](maps\\ko00220.html) |
| ko00340 | Histidine metabolism | 1/7 | 1/32 | 7/329 | 0.514828837 | 1 | MWSmce118 | C02835 | 4/102 | 4/792 | 102/24053 | 0.434032125 | 1 | Cluster-5709.0;Cluster-28617.10;Cluster-3363.0;Cluster-23150.1 | K00128+K00128+K00128+K00128 | [https:\\www.genome.jp\dbget-bin\www_bget?map00340](maps\\ko00340.html) |
| ko00908 | Zeatin biosynthesis | 1/5 | 1/32 | 5/329 | 0.402460428 | 1 | Zmfn000481 | C00190 | 3/75 | 3/792 | 75/24053 | 0.450554137 | 1 | Cluster-19592.0;Cluster-34264.6;Cluster-24307.6 | K23452+K13496+K13496 | [https:\\www.genome.jp\dbget-bin\www_bget?map00908](maps\\ko00908.html) |
| ko00310 | Lysine degradation | 1/13 | 1/32 | 13/329 | 0.742434227 | 1 | mws1346 | C00956 | 9/258 | 9/792 | 258/24053 | 0.477726224 | 1 | Cluster-34140.11;Cluster-5709.0;Cluster-28617.10;Cluster-3090.0;Cluster-3363.0;Cluster-13419.6;Cluster-5215.5;Cluster-26002.4;Cluster-23150.1 | K11420+K00128+K00128+K00382+K00128+K22748+K11420+K11420+K00128 | [https:\\www.genome.jp\dbget-bin\www_bget?map00310](maps\\ko00310.html) |
| ko01240 | Biosynthesis of cofactors | 1/34 | 1/32 | 34/329 | 0.974703629 | 1 | mws0133 | C00153 | 32/994 | 32/792 | 994/24053 | 0.578606175 | 1 | Cluster-14596.2;Cluster-2549.0;Cluster-4376.0;Cluster-2207.0;Cluster-1920.0;Cluster-5709.0;Cluster-3923.0;Cluster-28617.10;Cluster-428.0;Cluster-5250.0;Cluster-4785.0;Cluster-25728.0;Cluster-3890.1;Cluster-32502.5;Cluster-3090.0;Cluster-2360.0;Cluster-840.0;Cluster-570.0;Cluster-32502.0;Cluster-3363.0;Cluster-3037.0;Cluster-2758.0;Cluster-3600.0;Cluster-35212.1;Cluster-822.0;Cluster-2442.0;Cluster-956.0;Cluster-13732.1;Cluster-32695.0;Cluster-32730.4;Cluster-23150.1;Cluster-32470.6 | K00789+K00600+K03146+K02259+K14190+K00128+K03146+K00128+K02492+K00789+K00600+K01938+K00789+K00789+K00382+K03146+K00789+K12502+K00789+K00128+K01919+K01845+K00940+K00940+K06215+K06215+K08679+K00789+K01950+K03644+K00128+K03146 | [https:\\www.genome.jp\dbget-bin\www_bget?map01240](maps\\ko01240.html) |
| ko00943 | Isoflavonoid biosynthesis | 1/2 | 1/32 | 2/329 | 0.1853362 | 1 | Lmdn006025 | C12631 | 1/27 | 1/792 | 27/24053 | 0.595255864 | 1 | Cluster-20566.0 | K13260 | [https:\\www.genome.jp\dbget-bin\www_bget?map00943](maps\\ko00943.html) |
| ko00330 | Arginine and proline metabolism | 1/13 | 1/32 | 13/329 | 0.742434227 | 1 | ML10197929 | C00300 | 10/336 | 10/792 | 336/24053 | 0.671616774 | 1 | Cluster-11218.3;Cluster-5709.0;Cluster-36373.4;Cluster-28617.10;Cluster-2428.0;Cluster-3363.0;Cluster-24060.11;Cluster-3723.0;Cluster-23150.1;Cluster-31733.5 | K01611+K00128+K01426+K00128+K01611+K00128+K01426+K01611+K00128+K01426 | [https:\\www.genome.jp\dbget-bin\www_bget?map00330](maps\\ko00330.html) |
| ko00410 | beta-Alanine metabolism | 1/8 | 1/32 | 8/329 | 0.563044605 | 1 | MWS2413 | C01073 | 7/239 | 7/792 | 239/24053 | 0.675568779 | 1 | Cluster-5709.0;Cluster-28617.10;Cluster-2613.0;Cluster-4955.0;Cluster-3363.0;Cluster-21541.4;Cluster-23150.1 | K00128+K00128+K01580+K01580+K00128+K01431+K00128 | [https:\\www.genome.jp\dbget-bin\www_bget?map00410](maps\\ko00410.html) |
| ko00564 | Glycerophospholipid metabolism | 1/7 | 1/32 | 7/329 | 0.514828837 | 1 | mws0120 | C00670 | 14/477 | 14/792 | 477/24053 | 0.707158335 | 1 | Cluster-33604.5;Cluster-369.0;Cluster-28553.0;Cluster-1962.0;Cluster-2714.0;Cluster-3280.0;Cluster-827.0;Cluster-2376.0;Cluster-1321.0;Cluster-26135.3;Cluster-33368.0;Cluster-26068.7;Cluster-22472.2;Cluster-33604.13 | K01115+K05929+K18693+K13513+K01115+K01115+K01126+K05929+K01115+K00006+K01115+K00655+K13510+K01115 | [https:\\www.genome.jp\dbget-bin\www_bget?map00564](maps\\ko00564.html) |
| ko00300 | Lysine biosynthesis | 1/7 | 1/32 | 7/329 | 0.514828837 | 1 | mws1346 | C00956 | 1/45 | 1/792 | 45/24053 | 0.778664302 | 1 | Cluster-27606.0 | K12524 | [https:\\www.genome.jp\dbget-bin\www_bget?map00300](maps\\ko00300.html) |
| ko00592 | alpha-Linolenic acid metabolism | 1/4 | 1/32 | 4/329 | 0.337200133 | 1 | pmb2786 | C16326 | 6/233 | 6/792 | 233/24053 | 0.782783826 | 1 | Cluster-2280.0;Cluster-4226.0;Cluster-17333.0;Cluster-4032.0;Cluster-11158.1;Cluster-21082.1 | K18857+K00454+K10525+K10525+K05894+K05894 | [https:\\www.genome.jp\dbget-bin\www_bget?map00592](maps\\ko00592.html) |
| ko00591 | Linoleic acid metabolism | 1/11 | 1/32 | 11/329 | 0.681460247 | 1 | Lmbn005443 | C14765 | 1/63 | 1/792 | 63/24053 | 0.879017535 | 1 | Cluster-4226.0 | K00454 | [https:\\www.genome.jp\dbget-bin\www_bget?map00591](maps\\ko00591.html) |
| ko01250 | Biosynthesis of nucleotide sugars | 1/13 | 1/32 | 13/329 | 0.742434227 | 1 | Zmfn000481 | C00190 | 12/505 | 12/792 | 505/24053 | 0.907359979 | 1 | Cluster-2333.0;Cluster-4110.0;Cluster-3215.0;Cluster-13629.0;Cluster-5658.0;Cluster-3655.0;Cluster-956.0;Cluster-1885.0;Cluster-33410.0;Cluster-31443.5;Cluster-22138.0;Cluster-29540.0 | K00975+K01784+K12450+K13379+K12448+K00975+K08679+K00975+K08678+K00469+K12447+K01784 | [https:\\www.genome.jp\dbget-bin\www_bget?map01250](maps\\ko01250.html) |
| ko00520 | Amino sugar and nucleotide sugar metabolism | 1/12 | 1/32 | 12/329 | 0.713514562 | 1 | Zmfn000481 | C00190 | 13/639 | 13/792 | 639/24053 | 0.978909345 | 1 | Cluster-2333.0;Cluster-4110.0;Cluster-29989.0;Cluster-3215.0;Cluster-13629.0;Cluster-5658.0;Cluster-3655.0;Cluster-956.0;Cluster-1885.0;Cluster-33410.0;Cluster-22138.0;Cluster-29540.0;Cluster-18281.0 | K00975+K01784+K20547+K12450+K13379+K12448+K00975+K08679+K00975+K08678+K12447+K01784+K20547 | [https:\\www.genome.jp\dbget-bin\www_bget?map00520](maps\\ko00520.html) |
| ko00760 | Nicotinate and nicotinamide metabolism | 2/10 | 2/32 | 10/329 | 0.252547641 | 1 | mws0133;mws0675 | C00153+C00455 | 1/127 | 1/792 | 127/24053 | 0.985927439 | 1 | Cluster-32695.0 | K01950 | [https:\\www.genome.jp\dbget-bin\www_bget?map00760](maps\\ko00760.html) |
| ko04075 | Plant hormone signal transduction | 1/2 | 1/32 | 2/329 | 0.1853362 | 1 | Lmtn004049 | C06082 | 20/1139 | 20/792 | 1139/24053 | 0.999557453 | 1 | Cluster-4104.0;Cluster-1981.0;Cluster-24467.0;Cluster-21584.0;Cluster-14565.5;Cluster-14565.2;Cluster-1774.0;Cluster-31055.10;Cluster-2007.0;Cluster-14565.6;Cluster-2965.0;Cluster-22520.9;Cluster-2650.0;Cluster-169.0;Cluster-36638.0;Cluster-24444.7;Cluster-30388.5;Cluster-33959.6;Cluster-23848.0;Cluster-32963.10 | K14496+K14496+K14496+K14496+K14513+K14513+K13415+K14491+K14484+K14513+K14494+K14510+K13463+K13415+K13422+K12126+K14514+K14431+K13422+K14510 | [https:\\www.genome.jp\dbget-bin\www_bget?map04075](maps\\ko04075.html) |
| ko00970 | Aminoacyl-tRNA biosynthesis | 1/18 | 1/32 | 18/329 | 0.849519519 | 1 | mws0230 | C00188 | 3/363 | 3/792 | 363/24053 | 0.99955856 | 1 | Cluster-31524.0;Cluster-31524.6;Cluster-34789.6 | K01872+K01872+K01870 | [https:\\www.genome.jp\dbget-bin\www_bget?map00970](maps\\ko00970.html) |

| **Table S4. Conjoint analysis of DEGs and DAMs. （Store-3m_vs_Store-1m_common_KEGG）** | | | | | | | | | | | | | | | | |
| --- | --- | --- | --- | --- | --- | --- | --- | --- | --- | --- | --- | --- | --- | --- | --- | --- |
| **KEGG_map** | **Description** | **Rich_factor_meta** | **DiffRatio_meta** | **BgRatio_meta** | **P-value_meta** | **Adjusted P-value_meta** | **Index_meta** | **CID_meta** | **Rich_factor_gene** | **DiffRatio_gene** | **BgRatio_gene** | **P-value_gene** | **Adjusted P-value_gene** | **Index_gene** | **KO_gene** | **Hyperlink** |
| ko02010 | ABC transporters | 2/39 | 2/24 | 39/327 | 0.809349788 | 1 | mws0217;Zmdp000972 | C00037+C22040 | 5/408 | 5/63 | 408/24053 | 0.00430109 | 0.240861027 | Cluster-35887.9;Cluster-33978.6;Cluster-5362.4;Cluster-10836.7;Cluster-35887.7 | K05666+K08711+K08711+K08711+K05666 | [https:\\www.genome.jp\dbget-bin\www_bget?map02010](maps\\ko02010.html) |
| ko00944 | Flavone and flavonol biosynthesis | 2/15 | 2/24 | 15/327 | 0.302775531 | 1 | mws0913;MWSHY0104 | C12626+C03951 | 1/13 | 1/63 | 13/24053 | 0.033528043 | 1 | Cluster-28023.0 | K13080 | [https:\\www.genome.jp\dbget-bin\www_bget?map00944](maps\\ko00944.html) |
| ko00310 | Lysine degradation | 2/13 | 2/24 | 13/327 | 0.245553871 | 1 | mws1346;mws0217 | C00956+C00037 | 2/258 | 2/63 | 258/24053 | 0.146664094 | 1 | Cluster-35355.5;Cluster-35355.9 | K14157+K14157 | [https:\\www.genome.jp\dbget-bin\www_bget?map00310](maps\\ko00310.html) |
| ko00380 | Tryptophan metabolism | 1/14 | 1/24 | 14/327 | 0.663742282 | 1 | mws0005 | C00398 | 2/272 | 2/63 | 272/24053 | 0.159419922 | 1 | Cluster-24060.11;Cluster-24060.12 | K01426+K01426 | [https:\\www.genome.jp\dbget-bin\www_bget?map00380](maps\\ko00380.html) |
| ko00908 | Zeatin biosynthesis | 1/5 | 1/24 | 5/327 | 0.318584099 | 1 | Zmfn000481 | C00190 | 1/75 | 1/63 | 75/24053 | 0.178811423 | 1 | Cluster-9528.0 | K00279 | [https:\\www.genome.jp\dbget-bin\www_bget?map00908](maps\\ko00908.html) |
| ko00330 | Arginine and proline metabolism | 2/13 | 2/24 | 13/327 | 0.245553871 | 1 | ML10197929;mws0018 | C00300+C00750 | 2/336 | 2/63 | 336/24053 | 0.219848102 | 1 | Cluster-24060.11;Cluster-24060.12 | K01426+K01426 | [https:\\www.genome.jp\dbget-bin\www_bget?map00330](maps\\ko00330.html) |
| ko00520 | Amino sugar and nucleotide sugar metabolism | 2/12 | 2/24 | 12/327 | 0.217171518 | 1 | Lmqn000780;Zmfn000481 | C00043+C00190 | 3/639 | 3/63 | 639/24053 | 0.234541407 | 1 | Cluster-29771.5;Cluster-36094.6;Cluster-28069.14 | K13648+K00963+K12446 | [https:\\www.genome.jp\dbget-bin\www_bget?map00520](maps\\ko00520.html) |
| ko00970 | Aminoacyl-tRNA biosynthesis | 1/18 | 1/24 | 18/327 | 0.756000179 | 1 | mws0217 | C00037 | 2/363 | 2/63 | 363/24053 | 0.245962941 | 1 | Cluster-34970.10;Cluster-33901.0 | K01873+K01870 | [https:\\www.genome.jp\dbget-bin\www_bget?map00970](maps\\ko00970.html) |
| ko00630 | Glyoxylate and dicarboxylate metabolism | 1/12 | 1/24 | 12/327 | 0.605886351 | 1 | mws0217 | C00037 | 2/366 | 2/63 | 366/24053 | 0.248875245 | 1 | Cluster-30487.0;Cluster-22263.3 | K00284+K01638 | [https:\\www.genome.jp\dbget-bin\www_bget?map00630](maps\\ko00630.html) |
| ko00941 | Flavonoid biosynthesis | 1/16 | 1/24 | 16/327 | 0.713408266 | 1 | pmb3074 | C12208 | 1/117 | 1/63 | 117/24053 | 0.264785941 | 1 | Cluster-28023.0 | K13080 | [https:\\www.genome.jp\dbget-bin\www_bget?map00941](maps\\ko00941.html) |
| ko01250 | Biosynthesis of nucleotide sugars | 2/13 | 2/24 | 13/327 | 0.245553871 | 1 | Lmqn000780;Zmfn000481 | C00043+C00190 | 2/505 | 2/63 | 505/24053 | 0.382542245 | 1 | Cluster-36094.6;Cluster-28069.14 | K00963+K12446 | [https:\\www.genome.jp\dbget-bin\www_bget?map01250](maps\\ko01250.html) |
| ko00770 | Pantothenate and CoA biosynthesis | 1/7 | 1/24 | 7/327 | 0.416522554 | 1 | mws0018 | C00750 | 1/184 | 1/63 | 184/24053 | 0.383942846 | 1 | Cluster-20438.4 | K01598 | [https:\\www.genome.jp\dbget-bin\www_bget?map00770](maps\\ko00770.html) |
| ko00999 | Biosynthesis of various plant secondary metabolites | 2/17 | 2/24 | 17/327 | 0.359545803 | 1 | mws1389;pmb0064 | C17148+C04831 | 1/195 | 1/63 | 195/24053 | 0.401598589 | 1 | Cluster-29127.1 | K23136 | [https:\\www.genome.jp\dbget-bin\www_bget?map00999](maps\\ko00999.html) |
| ko00592 | alpha-Linolenic acid metabolism | 1/4 | 1/24 | 4/327 | 0.263888508 | 1 | pmb2786 | C16326 | 1/233 | 1/63 | 233/24053 | 0.458844175 | 1 | Cluster-34205.4 | K00232 | [https:\\www.genome.jp\dbget-bin\www_bget?map00592](maps\\ko00592.html) |
| ko00410 | beta-Alanine metabolism | 1/8 | 1/24 | 8/327 | 0.460283363 | 1 | mws0018 | C00750 | 1/239 | 1/63 | 239/24053 | 0.467376094 | 1 | Cluster-34205.4 | K00232 | [https:\\www.genome.jp\dbget-bin\www_bget?map00410](maps\\ko00410.html) |
| ko01240 | Biosynthesis of cofactors | 2/34 | 2/24 | 34/327 | 0.739792581 | 1 | mws0217;mws0018 | C00037+C00750 | 3/994 | 3/63 | 994/24053 | 0.485830583 | 1 | Cluster-36094.6;Cluster-36201.0;Cluster-20438.4 | K00963+K01465+K01598 | [https:\\www.genome.jp\dbget-bin\www_bget?map01240](maps\\ko01240.html) |
| ko00240 | Pyrimidine metabolism | 1/11 | 1/24 | 11/327 | 0.573493448 | 1 | MWSslk257 | C00055 | 1/280 | 1/63 | 280/24053 | 0.522237005 | 1 | Cluster-36201.0 | K01465 | [https:\\www.genome.jp\dbget-bin\www_bget?map00240](maps\\ko00240.html) |
| ko00480 | Glutathione metabolism | 2/7 | 2/24 | 7/327 | 0.086474706 | 1 | mws0217;mws0018 | C00037+C00750 | 1/334 | 1/63 | 334/24053 | 0.586084798 | 1 | Cluster-29869.11 | K00432 | [https:\\www.genome.jp\dbget-bin\www_bget?map00480](maps\\ko00480.html) |
| ko01200 | Carbon metabolism | 1/14 | 1/24 | 14/327 | 0.663742282 | 1 | mws0217 | C00037 | 3/1235 | 3/63 | 1235/24053 | 0.634333647 | 1 | Cluster-36622.3;Cluster-34205.4;Cluster-22263.3 | K01610+K00232+K01638 | [https:\\www.genome.jp\dbget-bin\www_bget?map01200](maps\\ko01200.html) |
| ko01100 | Metabolic pathways | 14/208 | 14/24 | 208/327 | 0.783609665 | 1 | pme0278;Lmdn006025;mws1389;ML10197929;MWSslk257;pmb0064;mws1346;MWSmce118;mws0217;mws0018;mws0005;Lmqn000780;Zmfn000481;mws0675 | C00666+C12631+C17148+C00300+C00055+C04831+C00956+C02835+C00037+C00750+C00398+C00043+C00190+C00455 | 22/9441 | 22/63 | 9441/24053 | 0.79694961 | 1 | Cluster-28023.0;Cluster-29820.5;Cluster-29771.5;Cluster-24060.11;Cluster-36094.6;Cluster-34268.5;Cluster-34300.9;Cluster-29767.1;Cluster-36622.3;Cluster-26724.0;Cluster-28069.14;Cluster-32848.2;Cluster-24060.12;Cluster-34205.4;Cluster-22263.3;Cluster-36201.0;Cluster-29869.11;Cluster-26817.1;Cluster-35355.5;Cluster-34361.4;Cluster-20438.4;Cluster-35355.9 | K13080+K05310+K13648+K01426+K00963+K01205+K05294+K18819+K01610+K03921+K12446+K03884+K01426+K00232+K01638+K01465+K00432+K09838+K14157+K01897+K01598+K14157 | [https:\\www.genome.jp\dbget-bin\www_bget?map01100](maps\\ko01100.html) |
| ko01110 | Biosynthesis of secondary metabolites | 9/117 | 9/24 | 117/327 | 0.50804851 | 1 | pme0278;Lmdn006025;mws1389;pmb3074;pmb0064;mws1346;mws0913;mws0217;mws0005 | C00666+C12631+C17148+C12208+C04831+C00956+C12626+C00037+C00398 | 9/4873 | 9/63 | 4873/24053 | 0.914572641 | 1 | Cluster-29127.1;Cluster-36094.6;Cluster-36622.3;Cluster-34205.4;Cluster-22263.3;Cluster-26817.1;Cluster-35355.5;Cluster-35355.9;Cluster-9528.0 | K23136+K00963+K01610+K00232+K01638+K09838+K14157+K14157+K00279 | [https:\\www.genome.jp\dbget-bin\www_bget?map01110](maps\\ko01110.html) |
